# Room-Temperature Powder-Form Lysine-Based Organogelators for Rapid Remediation of Oil-Spill-Contaminated Water

**DOI:** 10.3390/gels12100885

**Published:** 2026-10-01

**Authors:** Han-Qing Peng, Xiao-Yu Zhou, Shuai-Ke Liu, Ke-Lu Liu, Zuo-Zhao Zhai, Cai-Cai Zhang, Haitao Yu

**Affiliations:** 1 Hebei Key Laboratory of Organic Functional Molecules, College of Chemistry and Materials Science, Hebei Normal University, Shijiazhuang 050024, Chinazxy18766224905@stu.hebtu.edu.cn (X.-Y.Z.);; 2 College of Nationality, Hebei Normal University, Shijiazhuang 050091, China; 3 Department of Chemical and Environmental Engineering, Hebei Chemical &Pharmaceutical College, Shijiazhuang 050026, China; zhaizuozhao@hebcpc.edu.cn

**Keywords:** phase-selective organogelators, self-assembly, lysine-derived organogelators, water treatment, oil spill remediation

## Abstract

Oil spill remediation remains a major challenge in global marine pollution control. Given their rapid oil removal capability, reusability, and environmental compatibility, phase-selective organogelators (PSOGs) have become attractive candidates. Nevertheless, few powdered PSOGs have been reported to rapidly form phase-selective gels with crude oils. In this work, we synthesized a series of lysine-derived organogelators (**Lys**-**OUAs**) through a simple route and evaluated their gelation behavior. In particular, **Lys-OUA-C14** showed superior gelation ability and formed stable gels with a wide range of petroleum hydrocarbons. Results obtained from Fourier-transform infrared spectroscopy, UV-vis absorption spectroscopy, scanning electron microscopy, concentration-dependent and temperature-dependent ^1^H NMR, differential scanning calorimetry, and X-ray diffraction indicated that van der Waals forces, hydrogen bonding, and π–π stacking interactions were involved in the self-assembly of **Lys-OUA-C14**. Notably, **Lys-OUA-C14** rapidly solidified diesel and enabled efficient recovery within 120 s through three-dimensional networks, without the need for external heating/cooling cycles or co-solvents. Powdered **Lys-OUA-C14** can be readily synthesized in high yield and exhibits good oil removal efficiency, outstanding recyclability, and eco-friendliness, making it a promising material for large-scale remediation of oil-spill-contaminated water.

## 1. Introduction

With the rapid advancement of the global industrial economy, fossil fuels have become indispensable as core energy sources in both industrial production and daily life [1]. However, oil spill pollution arising from the full industrial chain, including exploration, transportation, and refining, has emerged as a severe global environmental challenge [2]. From the 2010 Gulf of Mexico oil incident, which discharged 210 million gallons of crude oil and claimed 11 lives, triggering a large-scale marine ecological disaster, to the 2020 incident of the Japanese vessel *Wakashio*, which leaked 1000 tons of fuel oil near Mauritius and inflicted irreversible damage on coral reef ecosystems, such oil spill events have not only resulted in enormous economic losses but also posed long-term threats to aquatic habitats, food chains, and the global ecological balance [3,4,5]. Most recently, a major oil spill occurred in the Kerch Strait (Black Sea) in December 2024, when two fuel tankers sank in a storm and released approximately 3000 tons of heavy mazut oil. This incident resulted in the loss of one crew member and contaminated over 60 km of vulnerable coastline. The disaster devastated coastal wetlands and seabird populations, endangered marine life, and left persistent hydrocarbon contamination that will threaten fisheries and biodiversity for decades [6]. Such recurrent accidents underscore the critical need for high-performance, environmentally compatible materials and technologies capable of rapid and effective oil spill response and remediation. Current mainstream oil spill treatment technologies include in situ burning, bioremediation, adsorptive solidification, and chemical dispersants [7,8,9,10,11,12]. Nevertheless, most of these approaches suffer from notable limitations. In situ burning tends to cause air pollution; bioremediation involves a long cycle and lacks selectivity; traditional adsorbent materials show low oil removal efficiency and insufficient oil recovery capacity; and chemical dispersants may induce secondary pollution. Given these limitations, none of these approaches can adequately fulfill the demand for rapid, efficient, and environmentally friendly emergency oil spill treatment. Consequently, the development of novel high-performance oil spill treatment technologies and materials for fast solidification and recovery of spilled oil has become a critical priority in the field of ecological and environmental protection [13].

Low-molecular-weight organogelators (LMOGs) represent a family of supramolecular soft materials that self-assemble into three-dimensional network structures through non-covalent interactions, including van der Waals forces, hydrogen bonding, π–π stacking, and hydrophobic effects, offering new strategies for oil spill pollution remediation [14,15]. Specifically, the advent of phase-selective organogelators (PSOGs) has allowed the selective gelation of the organic liquid from a biphasic mixture [16]. Since the first demonstration of phase-selective organogelators (PSOGs) for oil spill remediation by Bhattacharya and co-workers in 2001 [17], significant progress has been made in this field [18,19]. Distinguished by their phase selectivity, PSOGs can form mechanically stable organogels that selectively gelate the oil phase without affecting the aqueous phase, thereby confining spilled oil and preventing secondary diffusion. The resulting oil gel can be readily recovered by simple mechanical collection, and the gelator itself is recyclable, thus offering environmental compatibility and avoiding secondary pollution [20]. However, most reported PSOGs rely on heating–cooling cycles or co-solvent addition to achieve gelation, conditions that are impractical for real-world oil spill response. Obviously, heating large volumes of seawater is unfeasible, and the widespread application of co-solvents for large-scale remediation would impose unacceptable environmental and economic burdens. Therefore, the direct utilization of PSOGs in the solid state at ambient temperature has become a research focus in marine oil spill remediation, with significant advances achieved in the treatment of refined petroleum products and crude oil in recent years. Sureshan and co-workers developed glucose-derived PSOGs that can be directly applied as fine powders [21]. Chaudhuri’s group developed naphthalene diimide-based gelators capable of gelling heavy crude oil almost instantaneously in water [22]. Meanwhile, Zeng demonstrated that a wetting strategy could substantially enhance the gelation rate [23]. Very recently, we reported a benzimidazole-based gelator for oil spill treatment in solid form, which exhibited gelation ability toward a broad spectrum of organic solvents and fuel oils [24]. Nevertheless, the synthetic procedure for benzimidazole-based gelators tends to be somewhat cumbersome, inevitably involving significant solvent consumption and excessive energy input. Such drawbacks greatly restrict their practical applicability, particularly for emergency oil spill remediation scenarios that require rapid on-site deployment. Therefore, it remains a critical and challenging research topic to exploit advanced PSOGs featuring facile synthesis, direct solid-state application, rapid phase-selective gelation capability, and favorable biocompatibility. Given these combined advantages, the development of advanced gelators with such features represents a critical and challenging research priority, both in fundamental science and for practical environmental remediation.

Given the application potential of organogelators based on PSOGs, we designed and synthesized a series of lysine-derived gelators (**Lys-OUAs**) through a simple one-step synthetic procedure. Using lysine as the core skeleton, hydrophobic long alkyl chains and functional groups were incorporated to modulate the strength and spatial arrangement of intermolecular non-covalent interactions. Notably, these gelators can be directly applied as powders to oil–water mixtures, initiating rapid gelation without any external triggers (e.g., heating, cooling, or co-solvents). They demonstrated remarkable gelation performance toward diverse petroleum hydrocarbons, including crude oil, diesel, and kerosene. Among them, **Lys-OUA-C14** exhibited outstanding performance for the remediation of diesel and crude oil from oil–seawater biphasic systems. This gelator is readily synthesized via a straightforward one-step route, affording the target product in high yield without tedious post-purification. As a result, **Lys-OUA-C14** requires a low gelation dosage (1.59 wt% for diesel, 0.34 wt% for crude oil), confers outstanding mechanical stability on the resulting organogels, and achieves rapid gelation (diesel within 120 s, crude oil within 10 min). In terms of practical application, the oil removal procedure is remarkably straightforward and operationally simple, requiring nothing more than direct sprinkling of the gelator powder onto the oil-contaminated area. Moreover, it achieves a high oil removal efficiency of over 95%, while also exhibiting favorable reusability (> 90% after five cycles) and inherent eco-friendliness for aquatic environments. The innovation of this work therefore resides in integrating direct powder application, rapid phase-selective gelation, high oil recovery, and good reusability into a single lysine-based organogelator, providing a practical and environmentally benign strategy for remediating oil-spill-contaminated water.

## 2. Results and Discussion

Non-covalent interactions are vital for the formation and stabilization of supramolecular gels, serving as the fundamental driving force for the self-assembly of low-molecular-weight organic gelators (LMOGs) into three-dimensional network structures. By incorporating suitable functional groups into the molecular backbone, LMOGs can be rationally endowed with the ability to gelate specific solvents through synergistic non-covalent interactions, such as π–π stacking, hydrogen bonding, van der Waals forces, hydrophobic interactions, and electrostatic interactions. Consequently, the rational design and synthesis of gelators containing tailored functional groups are crucial for the advancement of functional supramolecular gel materials [25,26,27]. In this work, a series of **Lys-OUAs** was designed and synthesized using L-lysine as the core scaffold (Scheme 1). As essential natural building blocks of living organisms, L-amino acids possess numerous inherent advantages that make them ideal scaffolds for gelator design, including excellent biocompatibility, non-toxicity, environmental friendliness, wide availability, and cost-effectiveness. Furthermore, the amino and carboxyl groups in their molecular skeletons provide versatile reactive sites for the introduction of various functional moieties, facilitating the design of organic gelators with tunable gelation properties [28,29].

### 2.1. Gelation Capability of Lys-OUAs

The gelation performance of the synthesized **Lys-OUAs** was initially evaluated in a range of organic liquids and oils using the “inversion-vial” method. The gelation behavior of the eight compounds was assessed in diverse polar and nonpolar organic solvents as well as fuel oils through heating–cooling cycles. The gelation abilities and critical gelation concentrations (CGCs) of the **Lys-OUAs** series are summarized in Tables S1 and S2 (see Supporting Information). These results reveal that compounds containing long alkyl chains (A1–A4) generally gelated a wider variety of oils than those without long alkyl chains (A5–A8) [30]. We attribute this to the strengthened van der Waals interactions of the gelator with the oil, which facilitate rapid dispersion of the gelator in the oily medium. Subsequently, intermolecular hydrogen bonding, π–π stacking, and van der Waals forces drive the association of gelator molecules into a three-dimensional network, effectively encapsulating the oil components within the network cavities. Since the **Lys-OUA** series shares a common scaffold, the alkyl chain length is the primary structural variable and thus the main tunable parameter for modulating gelation behavior. Gelation performance improved with increasing alkyl chain length, indicating that the introduction and precise control of the alkyl chain length play a crucial role in regulating the gelation behavior in organic liquids. Hydrogen bonding and π–π stacking provide the fundamental driving forces for network formation, while the alkyl chain mainly governs van der Waals interactions and hydrophobicity, which control solubility and dispersion in the oil phase. In particular, **Lys-OUA-C14** displayed superior gelation performance among all derivatives and was thus chosen as the representative supramolecular gelator for further detailed investigation. A detailed summary of the gelation abilities, such as CGC/BCGC and gel-to-sol transition temperatures (*T*_gel_) for **Lys-OUA-C14**, is provided in Tables S3 and S4 (see Supporting Information).

The gelation capability of **Lys-OUA-C14** was further evaluated toward a variety of long-chain alkanes (*n*-hexane, *n*-heptane, *n*-dodecane, and *n*-tetradecane), as well as petroleum ether, diesel, gasoline, crude oil, and kerosene (Figure 1a). The superior gelation ability of **Lys-OUA-C14** over other shorter-chain analogs is primarily attributed to its elongated alkyl chains, which enhance van der Waals interactions with the oil phase, thereby accelerating dispersion of the gelator and promoting self-assembly into stable three-dimensional networks [31]. Structure–property analysis indicates that the length of the alkyl chain linked to the amide moiety plays a decisive role in regulating gelation performance. In addition to van der Waals forces, the amide groups contribute to network formation via intermolecular hydrogen bonding, while π–π stacking interactions between the benzene rings further reinforce the overall supramolecular architecture [32]. Moreover, the gels formed by **Lys-OUA-C14** exhibit excellent thermoreversibility upon repeated heating–cooling cycles (Figure 1b,c). Most of these gels also demonstrate long-term stability, with no obvious structural degradation observed after being stored in sealed vessels at room temperature for several weeks.

### 2.2. Self-Assembly Process of Lys-OUA-C14

Morphological analysis of the organogels formed by **Lys-OUA-C14** was performed using field-emission scanning electron microscopy (FE-SEM). To prepare xerogel samples for FE-SEM observation, the resulting organogels were first frozen in liquid nitrogen and subsequently lyophilized for 48 h to preserve the intrinsic supramolecular network structure. As observed in Figure 2, SEM revealed that the xerogels derived from **Lys-OUA-C14** in various organic solvents all exhibited well-defined three-dimensional (3D) network nanostructures. Solvent molecules are effectively trapped and immobilized within such networks by capillary and surface tension forces, thus facilitating the transformation from liquid to gel. Notably, the self-assembly behavior of **Lys-OUA-C14** was highly dependent on the solvent properties, leading to distinct aggregate morphologies. For instance, **Lys-OUA-C14** gels formed in toluene (Figure 2a,b) displayed dense and entangled fibrous aggregate structures, which can be attributed to π–π stacking between the solvent and the benzene rings of the gelator, directing one-dimensional fibrous growth. In contrast, in aliphatic *n*-hexane and *n*-heptane, where such gelator–solvent π–π interactions are absent, gelator–gelator interactions dominated by amide hydrogen bonding and van der Waals forces prevail, yielding lamellar and ribbon-like structures, respectively (Figure 2c–f). These observations indicate that the solvent does not merely act as a medium but intrinsically tunes the balance between gelator–gelator and gelator–solvent interactions, thereby determining the final assembly pathway and morphology.

To obtain insight into the driving forces for gelation, FT-IR spectra of **Lys-OUA-C14** were recorded for the neat solid state and for xerogels derived from different solvents. As presented in Figure 3, the neat solid **Lys-OUA-C14** exhibited characteristic absorption bands at 3311.17 cm^−1^ and 1542.29 cm^−1^, assigned to the stretching and bending modes of the amide N-H groups, respectively. These bands shifted to 3297.68 cm^−1^ and 1537.95 cm^−1^ in the diesel xerogel and to 3298.64 cm^−1^ and 1535.06 cm^−1^ in the toluene xerogel. For the neat solid, the amide I bands of C=O stretching vibrations appeared at 1648.36 cm^−1^, shifting to 1645.95 cm^−1^ in the diesel xerogel and to 1644.50 cm^−1^ in the toluene xerogel. These observations support the participation of intermolecular hydrogen bonds in the self-assembly process [33,34]. The subtle differences in FT-IR absorption between the two xerogels indicate variations in hydrogen bonding strength within distinct self-assembled architectures formed in different solvent environments. Additionally, the asymmetric and symmetric stretching modes of the alkyl groups were observed at 2924.04 cm^−1^ and 2852.68 cm^−1^ for the neat solid, shifting to 2921.14 cm^−1^ and 2850.76 cm^−1^ in the diesel xerogel and 2920.18 cm^−1^ and 2849.79 cm^−1^ in the toluene xerogel, which indicates variations in van der Waals interactions among the alkyl chains in different solvents. On the basis of these results, intermolecular hydrogen bonding and van der Waals interactions are identified as the predominant driving forces for the formation of the supramolecular gel network of **Lys-OUA-C14**.

To further understand the intermolecular interactions driving gelation, we performed concentration-dependent ^1^H NMR experiments in a mixed solvent of DMSO-*d*_6_/CDCl_3_ (*v*/*v* = 1:1) to elucidate the effect of **Lys-OUA**-C14 concentration on molecular packing within the gel network. As illustrated in Figure S1a, the amide proton signals appeared at *δ* 7.55 and 6.91 ppm at low concentrations. With increasing concentration, these signals shifted downfield to *δ* 7.61 ppm and 6.97 ppm, respectively, indicating the participation of hydrogen bonding between amide groups in the gelation process. In addition, the aromatic protons of the benzene ring exhibited a downfield shift from *δ* 7.29 ppm to *δ* 7.31 ppm upon increasing concentration, suggesting the existence of π–π stacking interactions. Meanwhile, the alkyl protons at *δ* 1.22 ppm and 0.84 ppm also shifted downfield to *δ* 1.24 ppm and 0.86 ppm, respectively, suggesting that van der Waals interactions between the alkyl chains are enhanced with increasing concentration [35,36]. These concentration-dependent NMR data confirm that hydrogen bonding, π–π stacking, and van der Waals interactions represent the main driving forces for the self-assembly of **Lys-OUA-C14** into supramolecular gel networks, which is fully consistent with the FT-IR results. This cooperative mechanism was further supported by temperature-dependent ^1^H NMR (Figure S1b), in which heating reversed the observed chemical shift variations, in agreement with the disassembly of the supramolecular network.

To further illustrate the self-assembly characteristics and supramolecular ordering of **Lys-OUA-C14** in the gel state, powder X-ray diffraction (PXRD) measurements were performed on **Lys-OUA-C14** xerogels. A broad diffraction peak was observed at 21.35° (d = 0.42) (Figure S2), which is generally attributed to the disordered alkyl chains, further confirming that van der Waals interactions serve as the primary driving force during xerogel formation. Detailed analysis revealed periodic XRD reflections in the low-angle region for the xerogel at 2*θ* = 2.38°, 4.80°, and 7.08°, which correspond to *d*-spacings of 3.71 nm, 1.84 nm, and 1.25 nm, close to the ratio of 1: 1/2: 1/3. The characteristic diffraction peak at 2*θ* = 24.68° (*d* = 0.36 nm) confirmed the existence of π–π stacking interactions between benzene rings, which further stabilized the spatial structure of the gel [37,38,39]. The lamellar *d*-spacing (3.71 nm) is larger than the estimated molecular length of a single **Lys-OUA-C14** (3.23 nm) molecule yet smaller than twice that length, suggesting an interpenetrating arrangement of the long alkyl chains within the lamellae (Figure S3a). Density functional theory calculations were employed to analyze intermolecular interactions between two molecules. The calculated distances of 0.45 nm for alkyl chains and 0.34 nm for benzene rings correspond well to experimental values (0.42 nm and 0.36 nm) derived from XRD results, verifying the presence of van der Waals forces and π–π stacking interactions (Figure S3b) [40,41]. To further investigate the self-assembly behavior of the gelator in the organogel state, UV-vis absorption spectroscopy was employed. As illustrated in Figure S4, the UV-vis absorption spectra of **Lys-OUA-C14** in *n*-heptane were recorded at varying gelator concentrations, ranging from the pre-gel state to the critical gelation concentration (CGC). With increasing concentration, the maximum absorption peak gradually increased in intensity and exhibited a red shift from 219 nm to 224 nm [42]. The red shift of the maximum absorption peak can be attributed to *J*-aggregation, which arises from head-to-tail π–π stacking among aromatic moieties of adjacent **Lys-OUA-C14** molecules. These results provide direct evidence for the involvement of π–π interactions during the gelation process.

### 2.3. Thermal Stability and Mechanical Properties of Gels

Supramolecular gel formation generally exhibits excellent thermoreversibility, transitioning from a gel to a sol upon heating and reverting to the original gel state upon cooling without loss of properties. The thermal stability of the as-prepared gels was further assessed by differential scanning calorimetry (DSC), offering quantitative information on the gel-to-sol and sol-to-gel phase transitions. As illustrated in Figure S5a, the gel formed by **Lys-OUA-C14** in *n*-hexane (5.5 wt %) exhibited a gel-to-sol melting temperature (*T*_m_) of 63.18 °C upon heating from 25 °C to 100 °C, which exceeded the freezing temperature (*T*_f_) of 61.29 °C. The difference between *T*_m_ and *T*_f_ reflects typical thermal hysteresis behavior, a well-documented characteristic of thermoreversible supramolecular self-assembled systems [43,44]. This mild hysteresis is particularly advantageous for practical oil spill recovery applications, as it ensures that **Lys-OUA-C14** maintains its structural integrity and gelation functionality under practical conditions, thereby enhancing the reliability and efficiency of the oil remediation process. Additionally, the thermal transition temperature (*T*_m_) increased with rising gelator concentration (Figure S5b), suggesting that gelator concentration markedly affects the thermal stability of the gels [45]. Thermogravimetric analysis (TGA) revealed that **Lys-OUA-C14** begins to decompose at 374.78 °C (Figure 4), a temperature substantially higher than the typical reduced-pressure distillation temperatures used for petroleum fraction recovery [46]. This finding demonstrates that the molecular structure of **Lys-OUA-C14** remains intact during the thermal recovery of oily substances, eliminating the risk of gelator degradation and secondary contamination, thus further validating its suitability for practical oil spill remediation applications [47].

For practical applications in marine oil spill remediation, floating gels must possess sufficient mechanical robustness to withstand turbulent wave action and enable facile recovery and isolation. To evaluate this property, the rheological behavior of **Lys-OUA-C14** organogels in diesel, kerosene, and crude oil was measured at a fixed strain of 0.01% at 25 °C. As presented in Figure 5, the storage modulus (G′) and loss modulus (G″) remained nearly independent of the applied stress below the critical strain region, exhibiting only minor variations in magnitude. This behavior indicates that the three-dimensional supramolecular networks remained intact and capable of withstanding substantial external stress. However, when the oscillatory stress exceeded a certain threshold, catastrophic failure of the gels occurred, as evidenced by the sharp drop in G′ and G″, accompanied by a crossover of viscoelastic signals (G′ < G″). Frequency-sweep measurements revealed that both G′ and G″ of the organogels exhibited weak frequency dependence over the range from 0.01 to 100 rad·s^−1^. Across the entire tested interval, G′ was consistently larger than G″, demonstrating a dominant elastic response characteristic of stable supramolecular gels with strong resistance to external deformation [48]. Similar rheological behavior was observed for **Lys-OUA-C14** in petroleum ether and *n*-hexane (Figure S6). This mechanical performance far exceeds the minimal requirement for gelation confirmed by the inversion-vial method, highlighting the excellent mechanical stability of the gels. Notably, **Lys-OUA-C14** displayed favorable mechanical characteristics for the investigated oily solvents, underscoring its versatile and efficient gelation ability toward a wide range of oily substances.

### 2.4. Phase-Selective Gelation and Recovery of Oil Spills

Accidental oil spills result in substantial economic losses and lead to irreversible environmental pollution and ecological damage. Therefore, the development of materials for phase-selective gelation of oil in seawater is of considerable practical and environmental significance. Experimental investigations revealed that neat **Lys-OUA-C14** powder can rapidly and efficiently solidify petroleum products without requiring heating–cooling cycles or co-solvent addition to achieve phase-selective gelation. Moreover, it exhibited a low critical gelation concentration (CGC) and a low critical gelation concentration in biphasic systems (BCGC), as shown in Table S4. Notably, the BCGC value was slightly higher than the corresponding CGC value, indicating that the gelator retains its ability to gel oil products even in aqueous environments. On this basis, the phase-selective gelation performance of **Lys-OUA-C14** was further evaluated in seawater/oil biphasic systems using crude oil or refined petroleum products.

We evaluated the biocompatibility and environmental safety of **Lys-OUA-C14** before assessing its practical performance as a phase-selective gelator for oil spill treatment, as low toxicity toward aquatic organisms is essential for practical marine applications. In the toxicity assessment, the freshwater loach Misgurnus anguillicaudatus (collected from the Hutuo River near Shijiazhuang) was selected as the test organism because it is easy to maintain and sensitive to pollutants [49,50]. First, 12 mg of **Lys-OUA-C14** powder was added to 30 mL of water, and a loach (ca. 0.5 g, 3 cm) was placed in the water. Over the 7-day continuous observation period, no mortality or obvious abnormal behavior was observed (Figure S7), indicating that **Lys-OUA-C14** exhibits negligible acute toxicity toward aquatic organisms. In addition, the water solubility of **Lys-OUA-C14** was examined by ^1^H NMR spectroscopy in D_2_O, with no discernible resonances attributable to the gelator (Figure S8), confirming that **Lys-OUA-C14** is essentially insoluble in water. Taken together, the results demonstrated the environmental safety of **Lys-OUA-C14** and its suitability for phase-selective gelation in aquatic environments.

In laboratory simulation tests, **Lys-OUA-C14** powder was directly dispersed onto the surface of seawater spiked with diesel (seawater sampled from the coastal area of Qingdao, Shandong Province). The diesel floating on seawater was rapidly gelated, and the resulting gel exhibited favorable mechanical strength, as demonstrated by its ability to bear the water load when the sealed vial was inverted (Figure 6a). More importantly, the solidified diesel gel was sufficiently rigid to be easily separated from seawater using a spatula (Figure 6b–d, Video S1). This facile phase separation is crucial for practical applications, enabling efficient collection of gelled oil from the surface of water, minimizing secondary contamination, and allowing the recovered oil to be reused.

To further validate the phase-selective gelation performance of **Lys-OUA-C14** for practical marine oil spill cleanup, a series of experiments were carried out in oil–seawater biphasic systems with varying diesel-to-seawater volume ratios. Considering the large volume discrepancy between spilled oil and seawater under practical spill circumstances, two oil–water volume ratios were selected to simulate realistic thin oil spill conditions, specifically 1:10 (1 mL diesel to 10 mL seawater) and 1:20 (1 mL diesel to 20 mL seawater). In each experiment, different dosages of **Lys-OUA-C14** powder were evenly sprinkled over the oil layer with gentle stirring to simulate seawater wave agitation. The gelation time was recorded once the formed gel achieved sufficient rigidity to be easily lifted out of the water with a spatula.

Subsequently, the resulting gel was extracted with petroleum ether, and the gelator was recovered directly by suction filtration. The recovered gelator was washed with petroleum ether for a minimum of three cycles, followed by drying. The resulting mixture of petroleum ether and desorbed diesel was collected in a flask for vacuum distillation. The diesel removal efficiency and gelator recovery rate were calculated using the gravimetric method (Figure 6e–h). As shown in Figure 7a,b, increasing the dosage of **Lys-OUA-C14** contributed to a shorter gelation time and higher oil removal efficiency. Notably, varying the diesel-to-seawater volume ratio exerted no significant effect on the gelation behavior of the gelator toward diesel. At a **Lys-OUA-C14** loading of 30 mg, the oil removal efficiency approached 96%, with the gelation time shortened to less than 120 s. These findings confirm the robust phase-selective gelation capability of **Lys-OUA-C14** under conditions that simulate real marine oil spill scenarios, further supporting its practical applicability.

To evaluate the efficiency and recyclability of **Lys-OUA-C14** in practical oil spill treatment, the reusability of the recovered gelator was further examined. At an oil/water ratio of 1:10 (1 mL diesel: 10 mL seawater), 30 mg of the recovered **Lys-OUA-C14** was evenly applied onto the oil layer in an oil–seawater biphasic system. The aforementioned procedures for determining diesel removal efficiency and gelator recovery rate were repeated for five consecutive cycles. As illustrated in Figure 7c and Table S6, the gelation rate of **Lys-OUA-C14** showed no significant decline after five consecutive reuse cycles, with the gelation time remaining within 120 s. Moreover, 24.61 mg of **Lys-OUA-C14** recovered after the fifth cycle still maintained an oil removal efficiency of over 90% (Figure S9). These results demonstrate that **Lys-OUA-C14** possesses outstanding reusability, which can effectively reduce material consumption costs in practical applications and further enhance its practical applicability.

Marine crude oil spills inflict severe and lasting damage on marine ecosystems, cause considerable economic losses, and create long-term remediation burdens. In contrast to refined petroleum products, crude oil poses a greater threat due to its content of carcinogenic polycyclic aromatic hydrocarbons (PAHs), its complex chemical composition, and its tendency to form stable emulsions. These features result in widespread contamination, prolonged environmental persistence and considerable challenges in effective remediation. Under laboratory-simulated marine conditions, **Lys-OUA-C14** powder was sprinkled onto the surface of crude oil-contaminated seawater. The crude oil floating on the seawater was completely gelated within 10 min, and the solidified oil was readily separated from the aqueous phase with a spatula, as illustrated in Figure 8 and Video S2. This rapid and efficient gelation demonstrates the potential of **Lys-OUA-C14** for emergency response to crude oil spills in marine environments.

To quantitatively evaluate the phase-selective gelation performance of **Lys-OUA-C14** for crude oil, systematic experiments were conducted following the same experimental protocol as used for diesel at oil-to-water volume ratios of 1:10 (1 mL crude oil: 10 mL seawater) and 1:20 (1 mL crude oil: 20 mL seawater), simulating realistic thin oil spill conditions. It should be noted that crude oil is a highly complex mixture comprising compounds with a wide range of molecular weights. The three-dimensional network of **Lys-OUA-C14** effectively entraps the crude oil phase, including its heavier fractions, while extremely high-molecular-weight residues may require higher gelator dosages or longer gelation times. When the gelator dosage reached 50 mg, the crude oil floating on seawater was completely gelled within 10 min, achieving an oil removal efficiency of 96.32% (Figure 9a,b and Figure S12). Moreover, the recovered gelator retained a high crude oil removal efficiency of 87.60% even after five consecutive reuse cycles (Figure 9c and Figure S13, Table S7), demonstrating good reusability comparable to that observed for diesel. These promising results highlight the great potential of **Lys-OUA-C14** for efficient crude oil spill remediation. Therefore, its high stability and recyclability position **Lys-OUA-C14** as a competitive candidate for developing efficient and sustainable oil spill treatment materials. To further evaluate the performance of **Lys-OUA-C14**, a comparison with PSOGs reported over the past decade was performed, and the results are summarized in Table S5. This comparison highlights the advantages of **Lys-OUA-C14**, particularly its room-temperature powder application without heating or co-solvents, rapid gelation, high removal efficiency, and excellent reusability.

Drawing on the gelation behavior, self-assembly driving forces, and oil removal performance of **Lys-OUA-C14** described above, a plausible mechanism for the remediation of oil-contaminated water is proposed. This mechanism involves four stages: phase-selective gelation, self-assembly, three-dimensional network immobilization, and gel recovery. Owing to the hydrophobic nature of the long alkyl chains, **Lys-OUA-C14** preferentially partitions into the oil phase. Within the oil phase, intermolecular non-covalent interactions, including hydrogen bonding, van der Waals forces, and π–π stacking, drive the formation of one-dimensional structures. These structures subsequently entangle and cross-link to form a three-dimensional network that immobilizes oil molecules via capillary forces and surface tension, thereby converting the liquid into a gel. The resulting gel, which is thermoreversible and mechanically robust, can be removed from the water surface by mechanical collection, after which the oil can be recovered by vacuum distillation and the gelator can be recovered from the residue. This mechanism is consistent with the low water solubility and gelation behavior of **Lys-OUA-C14,** and it explains the selective oil immobilization observed in the remediation tests. From a practical perspective, the cost of **Lys-OUA-C14** is favorable for large-scale application. The gelator is synthesized from low-cost L-lysine via a one-step, chromatography-free route with >90% yield, and the gelation dosage is low (1.59 wt% for diesel, 0.34 wt% for crude oil). Moreover, the gelator can be recovered and reused for at least five cycles with a removal efficiency of >90% for diesel and >87% for crude oil, further reducing material consumption. In addition, the storage stability of **Lys-OUA-C14** was evaluated by contact angle and moisture uptake measurements. Even at 97% RH, the maximum moisture uptake was only 1.31% (Figure S10), indicating a low water affinity [51]. The water contact angle was 125.4° (Figure S11), confirming the hydrophobic nature of the gelator surface [52]. Together with its high thermal stability, these properties help maintain good flowability during long-term storage.

## 3. Conclusions

In conclusion, we have designed and synthesized a series of lysine-based organogelators (**Lys-OUAs**) with diverse substituents via a one-step, chromatography-free route in yields exceeding 90%. These gelators are capable of gelating a wide range of organic liquids and oils. Notably, they exhibit phase-selective gelation toward oils in powder form at room temperature, with no requirement for heating–cooling cycles or co-solvents. Benefiting from its long alkyl chain structure, **Lys-OUA-C14** stands out as the most promising candidate, demonstrating broad phase-selective gelation adaptability to diverse oils and low critical gelation concentrations in both single-phase and oil/water biphasic systems (1.59 wt% for diesel and 0.34 wt% for crude oil). Beyond its remarkable gelation performance, **Lys-OUA-C14** offers multiple practical advantages, including low gelation dosage, high chemical and mechanical stability of the resulting gels, rapid gelation (diesel <120 s, crude oil <10 min), high oil recovery efficiency exceeding 95% (95.90% for diesel, 96.32% for crude oil), and satisfactory reusability (>90% for diesel and >87% for crude oil after five cycles). The self-assembly of **Lys-OUA-C14** is driven by cooperative hydrogen bonding, π–π stacking, and van der Waals interactions, as confirmed by FT-IR, NMR, XRD, DSC, SEM, and rheological measurements. These features render **Lys-OUA-C14** a highly effective and environmentally benign material for marine oil spill remediation. Furthermore, this work provides a new strategy for the development of functional supramolecular gel materials for practical environmental remediation.

## 4. Materials and Methods

### 4.1. Materials

Reagents and solvents of analytical grade were obtained from Energy Chemical Corporation (Shanghai, China) and used as received. The progress of each reaction was monitored by thin-layer chromatography (TLC). Diesel was purchased from China National Petroleum Corporation (CNPC), Shijiazhuang, China. Crude oil was purchased from Sinopec Shijiazhuang Refining & Chemical Company (Shijiazhuang, China). Seawater used in the experiments was collected from the coastal area of Qingdao, China.

### 4.2. Instruments

Nuclear magnetic resonance (NMR) spectra were recorded on a Bruker AV 400 spectrometer (400 MHz, Bruker Corporation, Billerica, MA, USA) with CDCl_3_ or DMSO-*d*_6_ as the solvent. Signal multiplicities are abbreviated as s (singlet), d (doublet), t (triplet), and m (multiplet). Chemical shifts (*δ*) are reported in ppm, and coupling constants (*J*) in Hz. High-resolution mass spectra (HRMS) were obtained on an Agilent 1290 Infinity II-6545 mass spectrometer (Agilent Technologies, Inc., Santa Clara, CA, USA). Fourier-transform infrared (FT-IR) spectra and UV-vis spectra were recorded on a Shimadzu FTIR-8900 spectrometer and a Shimadzu UV 2700 spectrophotometer (Shimadzu Corporation, Kyoto, Japan), respectively. Rheological measurements were performed on an Anton Paar MCR302 rheometer (Anton Paar GmbH, Graz, Austria). Thermal analysis was conducted using a NETZSCH DSC-214 Polyma (NETZSCH-Gerätebau GmbH, Selb, Germany) and a Shimadzu DTG-60A analyzer (Shimadzu Corporation, Kyoto, Japan). Powder X-ray diffraction (PXRD) patterns were measured on a Bruker D8 ADVANCE X-ray diffractometer (Bruker Corporation, Billerica, MA, USA). Morphologies were observed by FE-SEM (Hitachi S4800, Hitachi High-Tech Corporation, Tokyo, Japan) after freezing in liquid nitrogen.

### 4.3. Procedures for the Synthesis of Compounds (***1**–**8***)

*N,N*-di(benzyloxycarbonyl)lysine (1 equiv), *N,N*-diisopropylethylamine (DIPEA, 2 equiv), and 1-[bis(dimethylamino)methylene]-1H-1,2,3-triazolo [4,5-b] pyridinium 3-oxide hexafluorophosphate (HATU, 1.1 equiv) were dissolved in *N,N*-dimethylformamide (DMF). The mixture was stirred magnetically at ambient temperature for 30 min. Subsequently, eight different alkylamines (including 1,3-propanediamine) or arylamines were added to the reaction mixture, and the reaction was stirred at 50 °C for 24 h. After completion of the reaction, as confirmed by thin-layer chromatography (TLC), the mixture was poured into a cold dilute hydrochloric acid solution to precipitate the crude product. The obtained precipitate was washed with excess distilled water, recovered by filtration, and dried in an oven to give the corresponding products (compounds A1–A8) as white solids in yields above 90%. All prepared compounds were characterized by ^1^H NMR and ^13^C NMR spectroscopy and ESI-MS.

### 4.4. Gelation Test

#### 4.4.1. Gelation Ability of Lys-OUAs

The gelation ability of the **Lys**-**OUAs** was evaluated according to previously reported methods [53,54]. Typically, an amount of the gelator (2 mg) was introduced into a defined volume (20 µL) of organic solvent in a sealed gel tube. The mixture was allowed to stand until the gelator had fully dissolved, during which the viscosity of the organic phase gradually increased, eventually leading to complete gelation. For samples that did not dissolve spontaneously, gentle heating was applied to achieve complete dissolution, followed by cooling to ambient temperature. The resulting samples were classified into four categories: solutions, partial gels, complete gels, and precipitates. Gel formation was confirmed using the standard “inversion-vial” method. Samples that exhibited no flow and maintained a solid-like state after inversion were designated as complete gels. Those exhibiting slight flow were classified as partial gels. Samples that were fully dissolved but failed to form a gel were defined as solutions, while those showing no dissolution were categorized as precipitates. The gelation abilities and characteristics of the **Lys-OUAs** are summarized in Table S1.

The thermoreversibility of the gels was evaluated by repeated heating–cooling cycles between 25 °C and 80 °C at a rate of 1 °C·min^−1^. Each cycle consisted of heating the gel until it became a clear solution, followed by cooling to room temperature to allow gel reformation. At least five consecutive cycles were performed to verify the reversibility.

#### 4.4.2. Determination of Critical Gelation Concentrations (CGCs) and Biphasic Critical Gelation Concentrations (BCGCs)

The dilution method was employed to determine the critical gelation concentration (CGC) of the synthesized gelator in various organic solvents [55]. To begin with, a concentrated mixture was prepared by suspending 2.0 mg of the gelator in 20 μL of the target solvent within a sealed glass vial. The system was then gradually diluted by successive additions of small aliquots of the same solvent, with repeated heating–cooling cycles after each addition, until the gel state could no longer be sustained. The minimum concentration at which the system still sustained a self-supporting gel was recorded as the CGC, expressed in wt%. Each measurement was performed in triplicate, and the consistent value was taken as the final CGC. The biphasic critical gelation concentrations (BCGCs) were evaluated in oil–seawater mixtures containing various oils at a volume ratio of 1:10 (1 mL: 10 mL). The experimental procedure was analogous to that used for the measurement of CGCs.

#### 4.4.3. Measurement of Gel-to-Sol Transition Temperature (*T*_gel_)

The gel-to-sol transition temperature (*T*_gel_) of the gels was measured using a traditional falling-ball method [56,57]. At the critical gelation concentration (CGC), a glass ball (6 mg, 2 mm in diameter) was gently positioned on the top of the gel. The tube with the gel was subsequently heated at 1 °C·min^−1^ in a water bath until the ball fell to the bottom, marking the complete gel-to-sol transition. The temperature at which this transition took place was recorded as *T*_gel_. Each measurement was conducted at least three times to ensure reproducibility.

### 4.5. Moisture Absorption

**Lys-OUA-C14** powder was dried at 105 °C to constant weight before testing. For each relative humidity (RH), three portions (about 10 mg each) were accurately weighed (m_0_) into open weighing bottles. The bottles were placed in sealed desiccators containing saturated salt solutions: LiCl for 11% RH, MgCl_2_ for 33% RH, NaCl for 75% RH, and K_2_SO_4_ for 97% RH. The desiccators were maintained at 25 °C. The mass of each sample was recorded as m_t_. Moisture uptake was calculated as (m_t_ − m_0_)/m_0_ × 100%. Data are presented as mean ± standard deviation (*n* = 3). Moisture adsorption isotherms were plotted from the 5-day data.

### 4.6. Contact Angle Measurement

The surface wettability of **Lys-OUA-C14** was evaluated by water contact angle (WCA) measurements. The powder was pressed into a flat pellet (or uniformly coated on a clean glass slide) before testing. A pendant drop of deionized water (about 5 μL) was placed on the sample surface at room temperature. The contact angle was recorded with a contact angle goniometer (model JY-PHb, Chengde Jinhe Instrument Manufacturing Co., Ltd., Chengde, China).

## Figures and Tables

**Figure 1:**
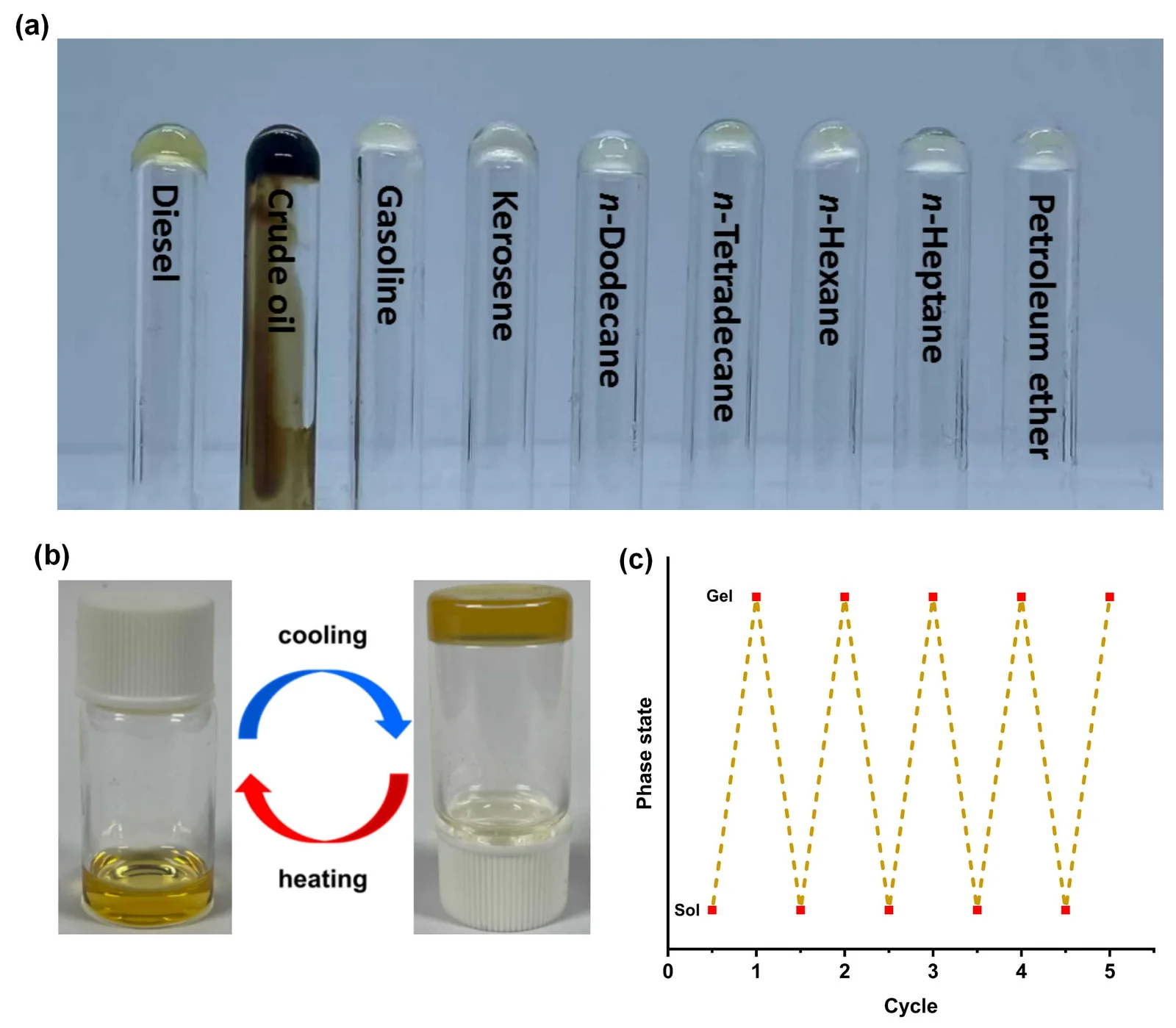
(**a**) The gelation behavior of **Lys-OUA-C14** in different solvents; (**b**) the thermoreversibility behavior of **Lys-OUA-C14** in diesel; (**c**) the gel-to-sol transition cycle.

**Figure 2:**
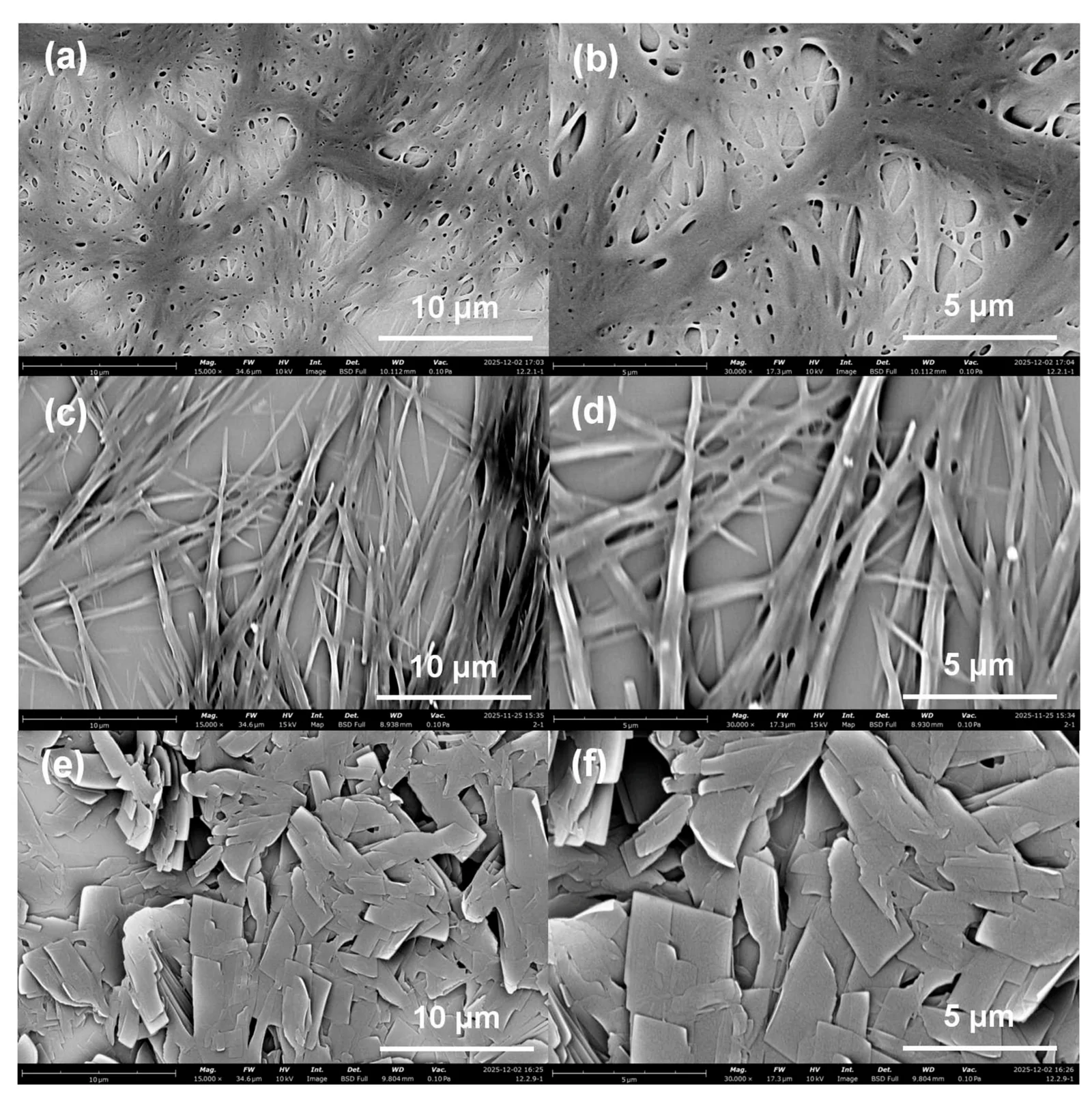
SEM images of **Lys-OUA-C14** (3.5 wt%) xerogels. (**a**,**b**) **Lys-OUA-C14**–toluene gel; (**c**,**d**) **Lys-OUA-C14**–*n*-heptane gel; (**e**,**f**) **Lys-OUA-C14**–*n*-hexane gel.

**Figure 3:**
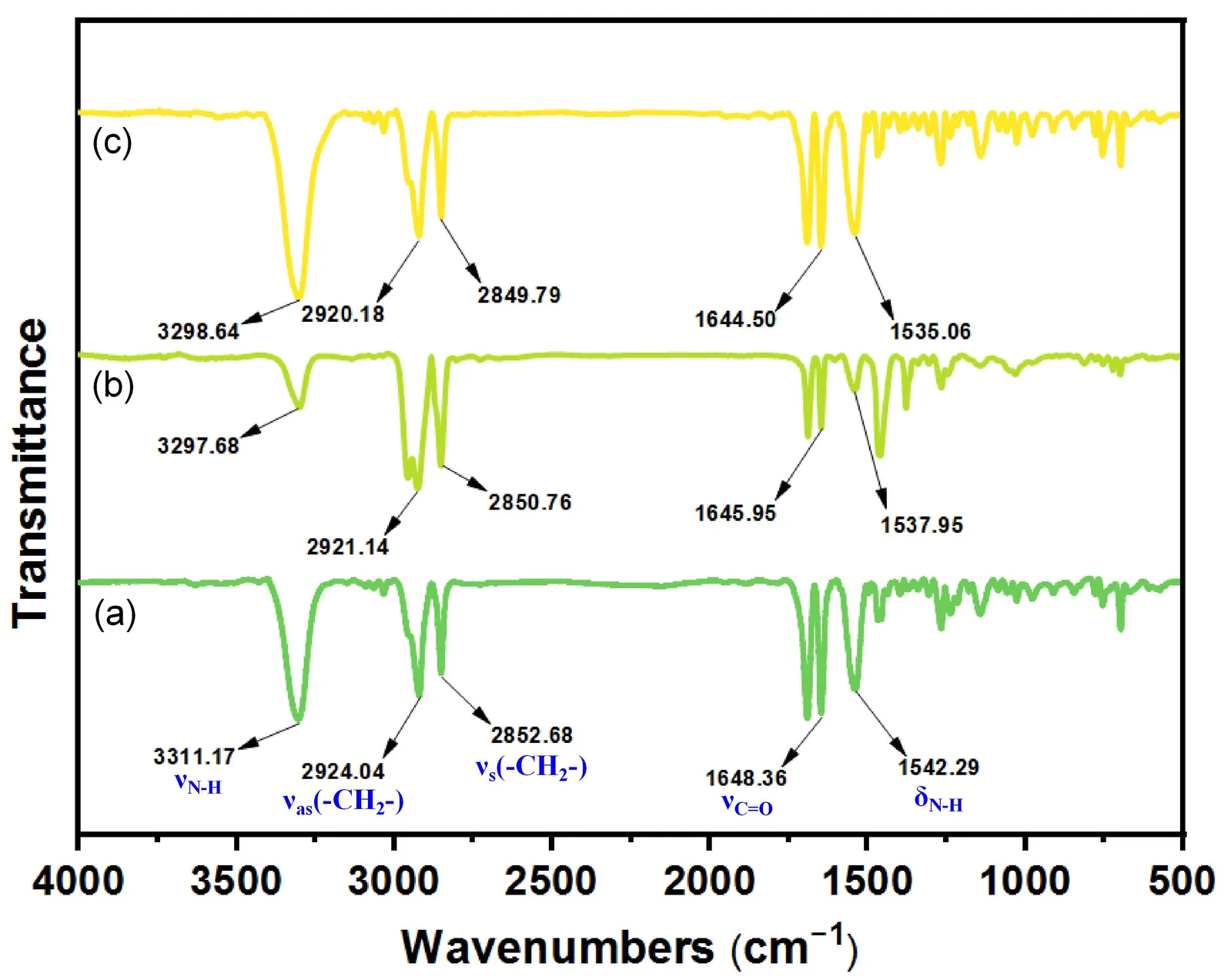
FT-IR spectra of **Lys-OUA-C14**: (**a**) neat solid; (**b**) xerogel from diesel at CGC; (**c**) xerogel from toluene at CGC.

**Figure 4:**
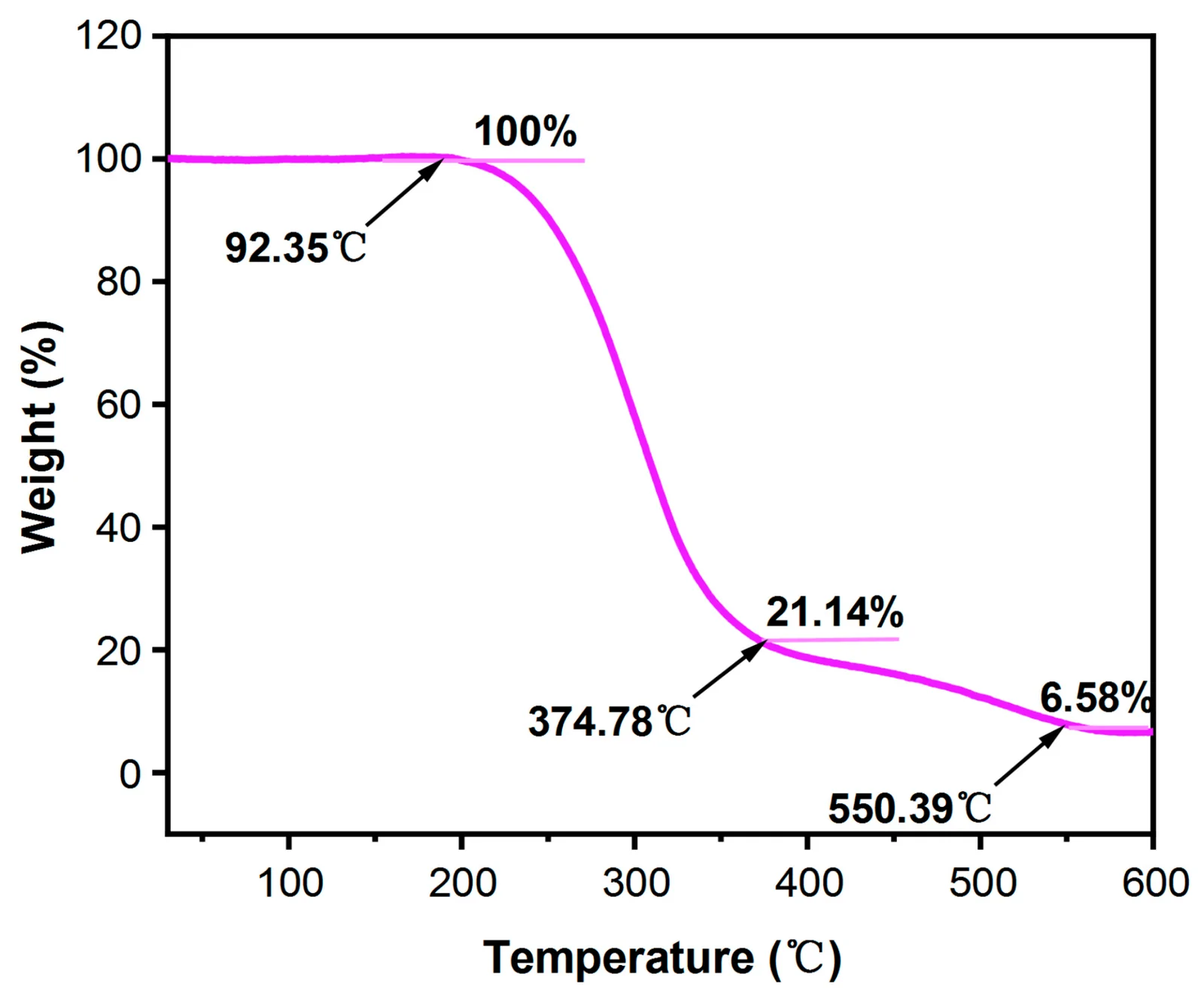
TGA curve of **Lys-OUA-C14** powder.

**Figure 5:**
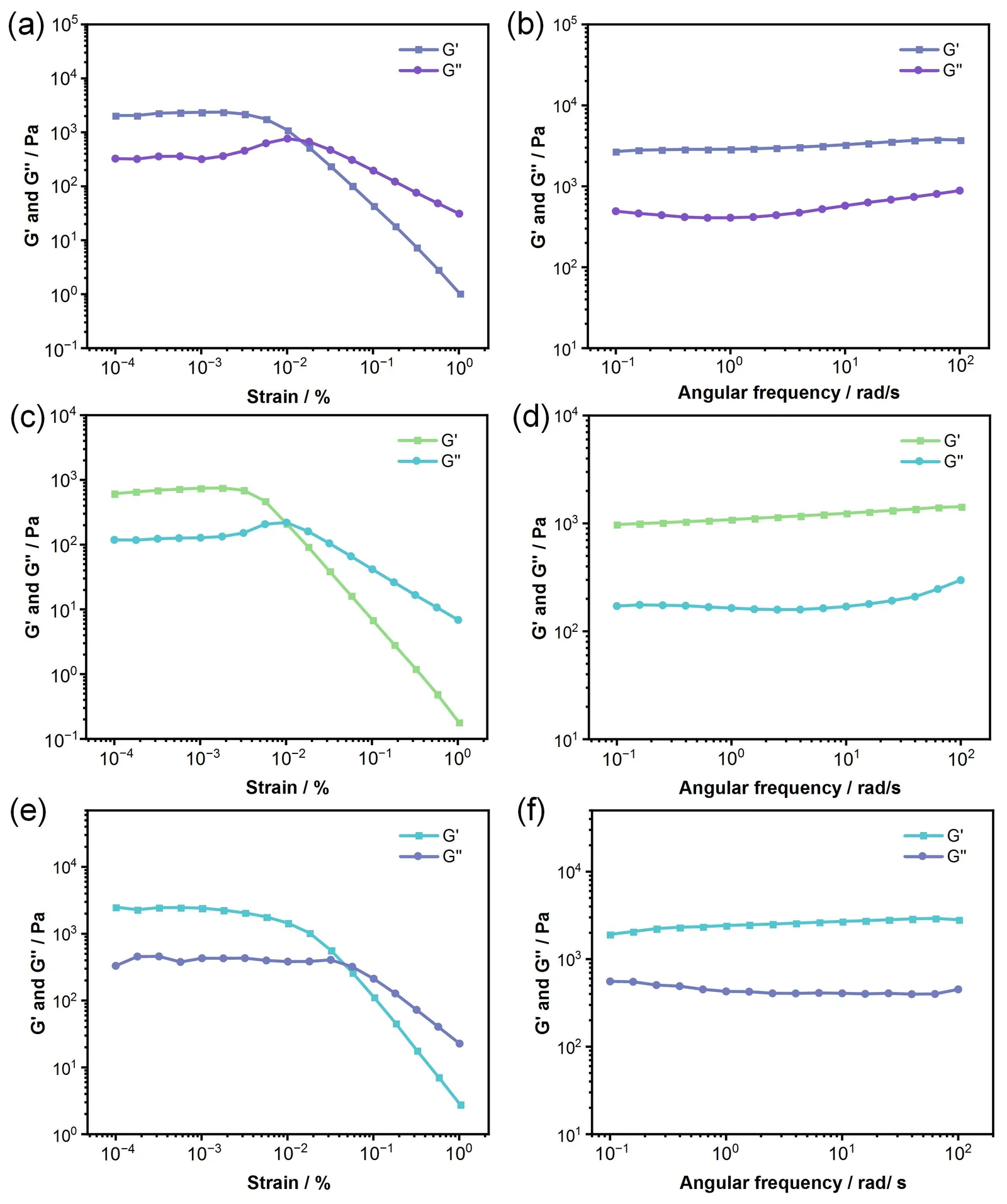
Rheological properties of **Lys-OUA-C14** (3.5 wt%) in diesel, kerosene, and crude oil: (**a**,**c**,**e**) stress sweeps; (**b**,**d**,**f**) frequency sweeps. G′ and G″ are the storage and loss moduli, respectively.

**Figure 6:**
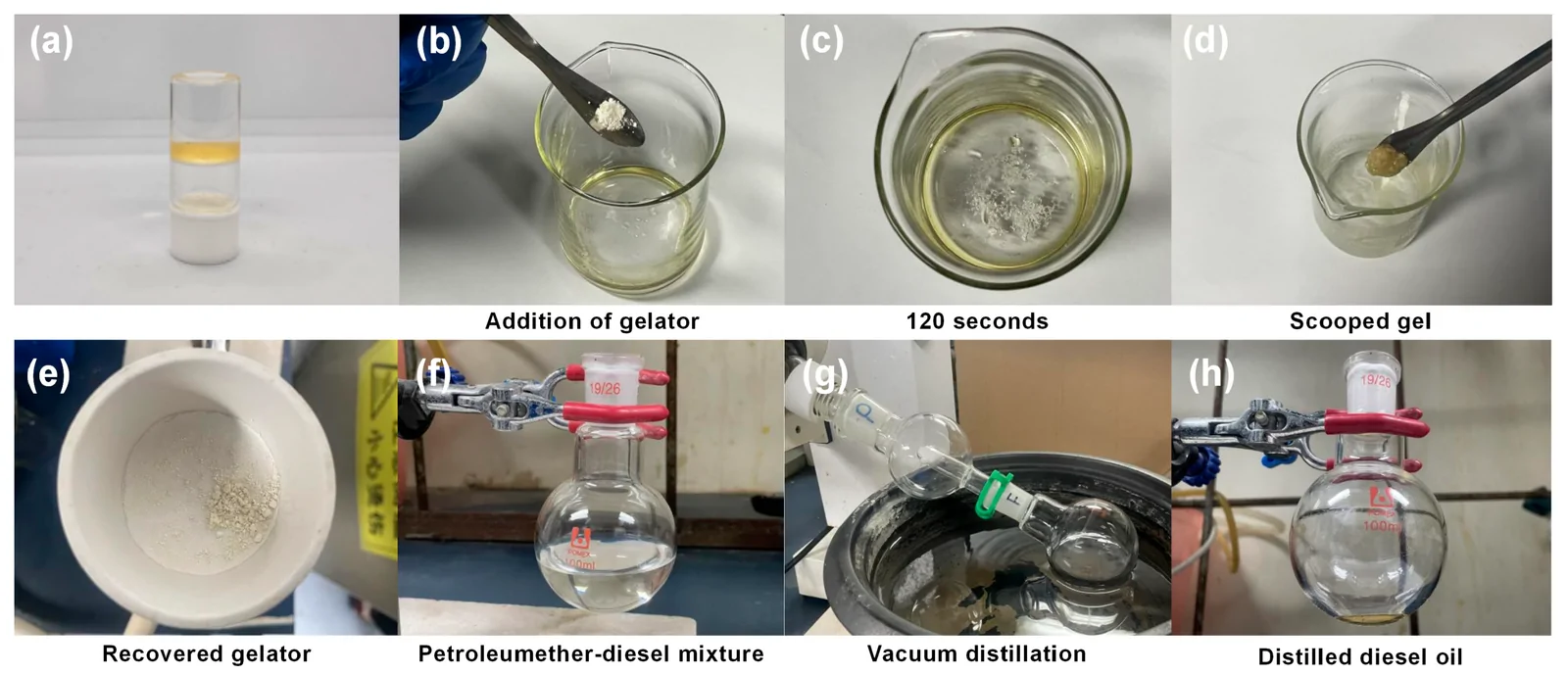
(**a**) Inverted vial of phase-selective diesel gel (0.3 mL diesel in 3 mL seawater) with **Lys-OUA-C14** at BCGC (2.38 wt%); (**b**–**d**) phase-selective gelation of diesel by **Lys-OUA-C14** powder (3.47 wt %, 30 mg) with a diesel/seawater ratio of 1:10 (1 mL: 10 mL); (**e**–**h**) demonstration of diesel recovery from the gel formed with **Lys-OUA-C14** as the phase-selective organogelator.

**Figure 7:**
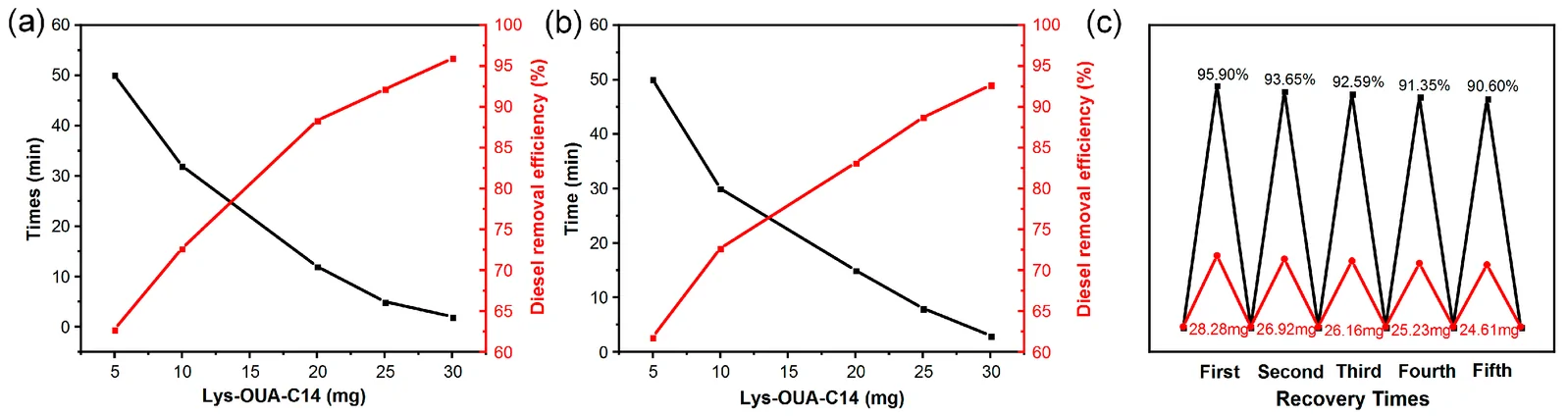
Gelation time and diesel removal efficiency of **Lys-OUA-C14** at diesel/seawater volume ratios of (**a**) 1:10 (1 mL:10 mL); (**b**) 1:20 (1 mL:20 mL) with different amounts of **Lys-OUA-C14**; (**c**) reusability of **Lys-OUA-C14** in diesel.

**Figure 8:**
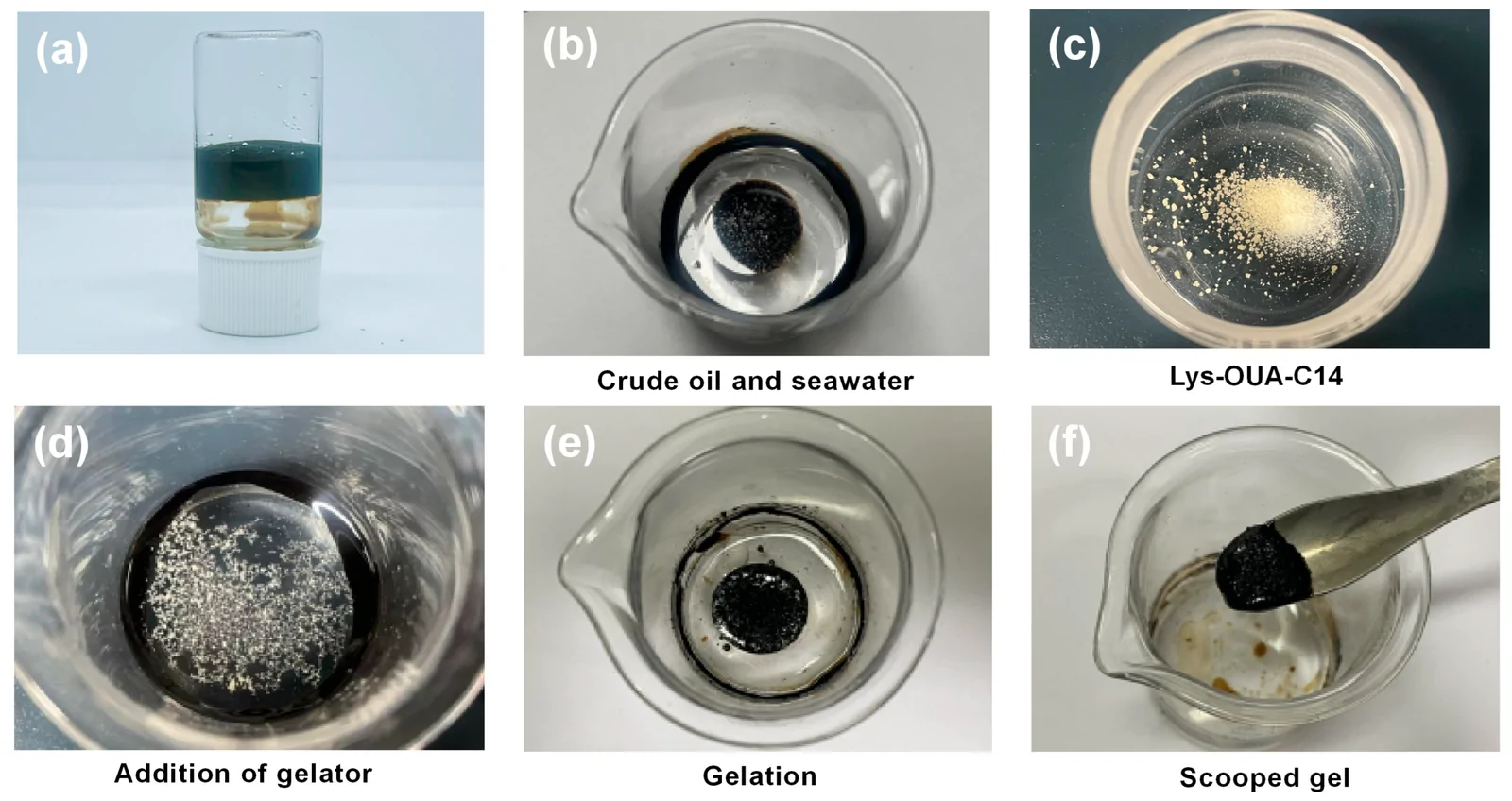
(**a**) Inverted vial of phase-selective crude oil gel (1 mL crude oil in 3 mL seawater) with **Lys-OUA-C14** at BCGC (2.54 wt%); (**b**–**f**) phase-selective gelation of crude oil using **Lys-OUA-C14** powder (4.49 wt%, 40 mg), with a crude oil/seawater volume ratio of 1:10 (1 mL:10 mL).

**Figure 9:**
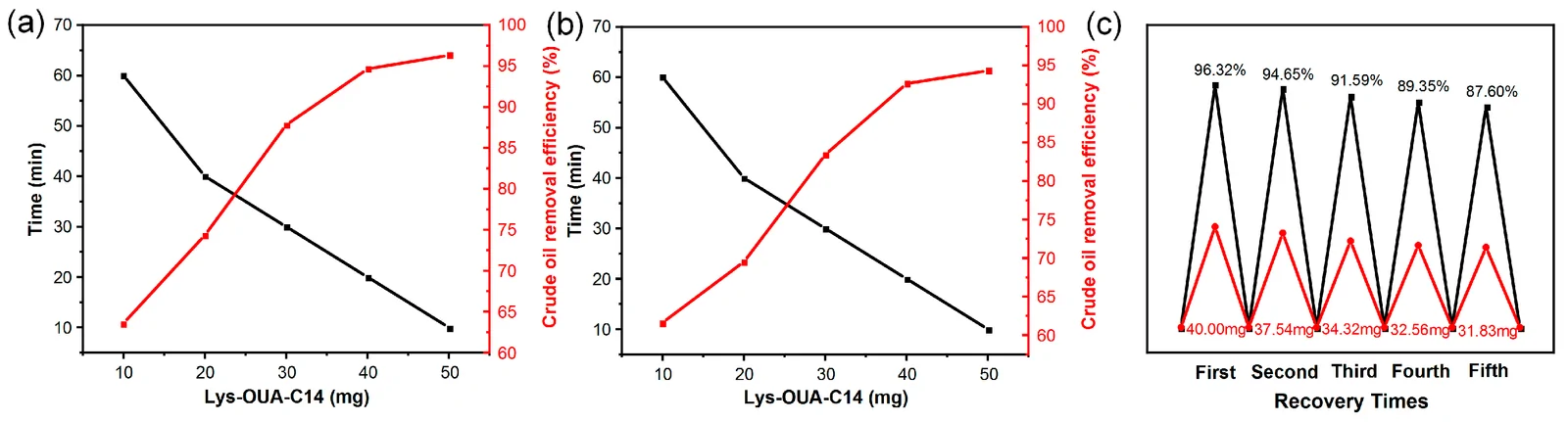
Gelation time and crude oil removal efficiency of **Lys-OUA-C14** at crude oil/seawater volume ratios of (**a**) 1:10 (1 mL:10 mL); (**b**) 1:20 (1 mL:20 mL) with different amounts of **Lys-OUA-C14**; (**c**) reusability of **Lys-OUA-C14** in crude oil.

**Scheme 1:**
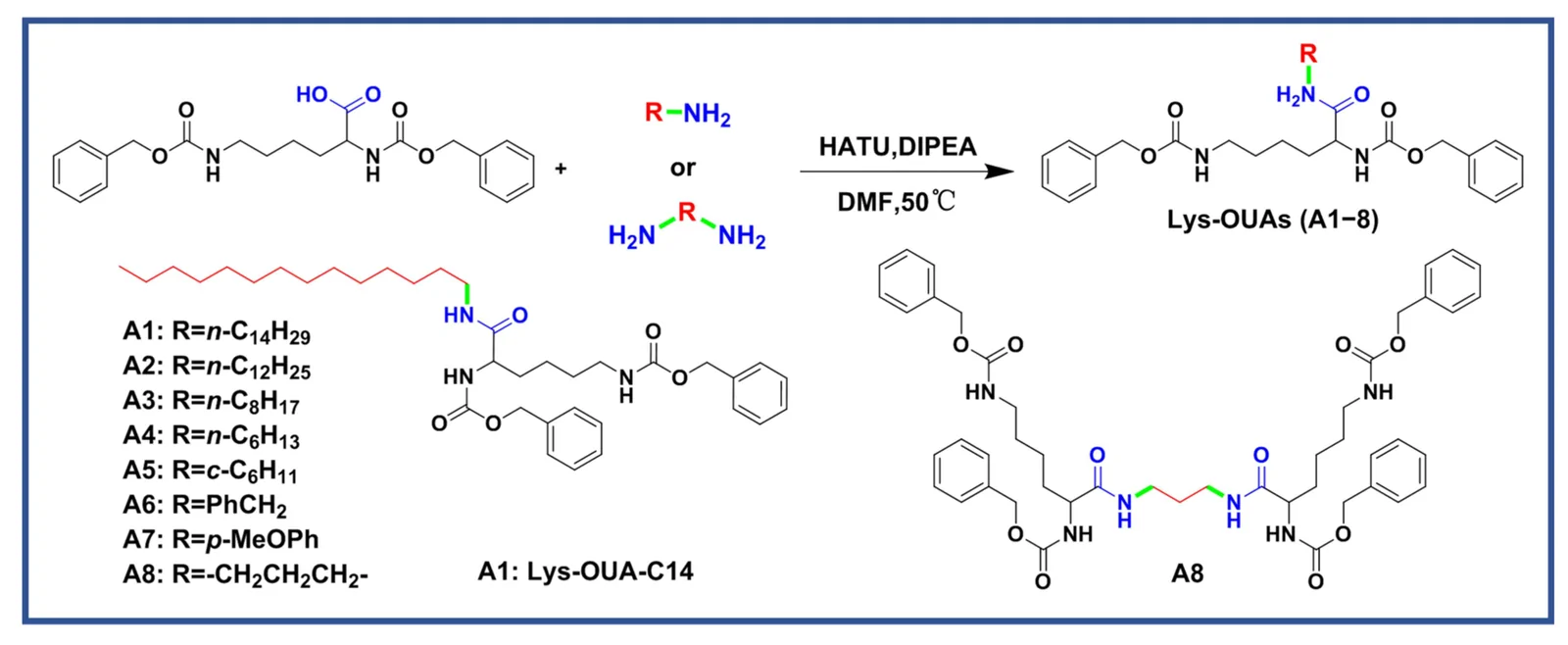
Synthetic routes of lysine-derived organogelators (**Lys-OUAs**).

## Data Availability

The original contributions presented in this study are included in the article. Further inquiries can be directed to the corresponding author.

## Supplementary Materials

The following supporting information can be downloaded at: https://www.mdpi.com/article/10.3390/gels12100885/s1, synthetic procedures for the **Lys-OUAs**; ^1^H NMR, ^13^C NMR and ESI-MS characterization; gelation capabilities in various liquids; FT-IR spectra; concentration-dependent ^1^H NMR and UV-vis spectra of **Lys-OUA-C14**; DSC thermograms; PXRD pattern; and reusability (PDF) [58,59,60,61,62,63,64,65,66].
