# Supplementary material for: Room-Temperature Powder-Form Lysine-Based Organogelators for Rapid Remediation of Oil-Spill-Contaminated Water
Source: Gels. 2026 Oct 1;12(10):885. doi: 10.3390/gels12100885 (PMC13704543; doi:10.3390/gels12100885)
Supplement: Supplementary file 1 [file gels-12-00885-s001.zip › gels-4560693-supplementary.pdf]

Supporting Information for

Room-Temperature Powder-Form Lysine-Based Organogelators for Rapid Remediation of Oil Spill Contaminated Water

Han-Qing Peng [a,b], Xiao-Yu Zhou [a], Shuai-Ke Liu [a], Ke-Lu Liu [a], Cai-Cai Zhang [a,*] and Haitao Yu [a,*]

[a] *College of Chemistry and Materials Science, Hebei Key Laboratory of Organic Functional Molecules, Hebei Normal University, Shijiazhuang, 050024, P. R. China.*

[b] *College of Nationality, Hebei Normal University, Shijiazhuang, 050091, P. R. China.*

*E-mail: caicaizhang@hebtu.edu.cn; haitaoyu@hebtu.edu.cn

CONTENTS	Page number	CONTENTS	Page number
Compound characterization	S2-S4	Figure S5	S14
Table S1	S4-S5	Figure S6	S14
Table S2	S5-S6	Figure S7	S15
Table S3	S6-S7	Figure S8	S15
Table S4	S7-S8	Figure S9	S16
Table S5	S8-S10	Figure S10	S16
Table S6	S11	Figure S11	S17
Table S7	S11	Figure S12	S17
Figure S1	S11-S12	Figure S13	S18
Figure S2	S12	Characterization (Figure S14-S37)	S19-S30
Figure S3	S13	References	S31-S32
Figure S4	S13		

Compound characterization

For clarity, the eight complex target molecular structures involved in this work are systematically labeled as A1–A8 throughout the text for concise description and discussion, and the corresponding full chemical names are matched one by one as follows: Lys-OUA-C14 (A1), Lys-OUA-C12 (A2), Lys-OUA-C8 (A3), Lys-OUA-C6 (A4), Lys-OUA-Cy (A5), Lys-OUA-Bn (A6), Lys-OUA-4-MeOPh (A7), and Lys2-PDA-BOC (A8), respectively.

Lys-OUA-C14 (A1)

white amorphous powder; Yield: 98.3%; ^{1}H NMR (400 MHz, DMSO-d_6, 298K) was as follows: δ 7.81 (t, *J* = 5.4 Hz, 1H), 7.34 (m, *J* = 4.1 Hz, 10H), 7.29 (m, *J* = 4.0 Hz, 1H), 7.22 (t, *J* = 5.1 Hz, 1H), 5.00 (d, *J* = 5.9 Hz, 4H), 3.88 (m, *J* = 13.7, 8.3 Hz, 1H), 2.97 (m, *J* = 18.0, 6.0 Hz, 4H), 1.55-1.47 (m, 2H), 1.36 (s, 2H), 1.23 (s, 22H), 0.85 (t, 3H). ^{13}C NMR (101 MHz, DMSO-d_6, 298K) δ 172.11, 156.45, 156.37, 137.75, 137.55, 128.81, 128.19, 128.15, 65.79, 65.57, 55.19, 38.88, 32.20, 31.79, 29.56, 29.51, 29.21, 26.78, 23.28, 22.59, 14.45. HR-ESI-MS (m/z): $C_{36}H_{55}N_3O_5$ $[M+H]^+$ calcd: 610.4220, found: 610.4230.

Lys-OUA-C12 (A2)

white amorphous powder; Yield: 93.6 %; ^{1}H NMR (400 MHz,DMSO-d_6, 298K) was as follows: δ 7.80 (t, *J* = 4.9 Hz, 1H), 7.34 (m, 10H), 7.30 (m, 1H), 7.21 (t, *J* = 4.3 Hz, 1H), 5.00 (d, *J* = 5.6 Hz, 4H), 3.89 (m, *J* = 12.8, 6.5 Hz, 1H), 3.03-2.95 (m, 4H), 1.54-1.47(m, 2H), 1.36 (s, 2H), 1.23 (s, 22H), 0.85 (t, 3H). ^{13}C NMR (101 MHz, DMSO-d_6, 298K) δ 172.11, 156.54, 156.37, 137.75, 137.55, 128.81, 128.18, 128.14, 65.79, 65.58, 55.20, 38.89, 32.21, 31.78, 29.55, 29.21, 26.78, 23.27, 22.58,14.43. HR-ESI-MS (m/z): $C_{34}H_{51}N_3O_5$ $[M+H]^+$ calcd: 582.3907, found: 582.3903; $[M+Na]^+$ calcd: 604.3726, found: 604.3725.

Lys-OUA-C8 (A3)

white amorphous powder; Yield: 95.2 %; ^{1}H NMR (400 MHz,DMSO-d_6, 298K) was as follows: δ 7.82 (t, *J* = 5.4 Hz, 1H), 7.38-7.34 (m, 10H), 7.32 (m, 1H), 7.23 (t, *J* = 5.0 Hz, 1H), 5.02 (d, *J* = 6.0 Hz, 4H), 3.91 (m, *J* = 13.3, 8.0 Hz, 1H), 3.09-3.01 (m, 2H), 2.99-2.95 (m, 2H), 1.56-1.52 (m, 2H), 1.39 (s, 2H), 1.25 (s, 14H), 0.88 (t, *J* = 6.2

Hz, 3H). ^{13}C NMR (101 MHz, DMSO-d_6, 298K) δ 172.11, 156.55, 156.37, 137.76, 137.55, 128.81, 128.20, 128.18, 128.13, 65.79, 65.57, 55.20, 38.89, 32.21, 31.70, 29.57, 29.48, 29.15, 26.77, 23.27, 22.57, 14.43. HR-ESI-MS (m/z): $C_{30}H_{43}N_3O_5$ $[M+H]^+$ calcd: 526.3281, found: 526.3277; $[M+Na]^+$ calcd: 548.3100, found: 548.3091.

Lys-OUA-C6 (A4)

white amorphous powder; Yield: 94 %; ^1H NMR (400 MHz,DMSO-d_6, 298K) was as follows: δ 7.81 (t, 1H), 7.34 (m, 10H), 7.28 (m, 1H), 7.22 (d, 1H), 5.00 (d, *J* = 5.6 Hz, 4H), 3.89 (m, *J* = 13.0, 7.9 Hz, 1H), 3.03-2.95 (m, 4H), 1.56-1.50 (m, 2H), 1.37 (s, 2H), 1.23 (s, 8H), 0.84 (d, *J* = 6.3 Hz, 3H). ^{13}C NMR (101 MHz, DMSO-d_6, 298K) δ 172.10, 156.55, 156.37, 137.76, 137.56, 128.81, 128.18, 128.14, 65.80, 65.58, 55.20, 38.91, 31.42, 29.57, 29.45, 26.43, 23.26, 22.52, 14.36. HR-ESI-MS (m/z): $C_{28}H_{39}N_3O_5$ $[M+H]^+$ calcd: 498.2968, found: 498.2964; $[M+Na]^+$ calcd: 520.2787, found: 520.2778.

Lys-OUA-Cy (A5)

white amorphous powder; Yield: 91.8 %; ^1H NMR (400 MHz, DMSO-d_6, 298K) was as follows: δ 7.67 (d, *J* = 7.7 Hz, 1H), 7.34 (m, *J* = 7.8 Hz, 10H), 7.26 (m, 1H), 7.21 (m, 1H), 5.00 (d, *J* = 6.2 Hz, 4H), 3.90 (m, *J* = 13.5, 7.7 Hz, 1H), 3.49 (m, *J* = 8.1 Hz, 1H), 2.95 (m, *J* = 11.3, 6.0 Hz, 3H), 1.67 (m, *J* = 8.0 Hz, 4H), 1.52 (t, 2H), 1.40-1.34 (t, 2H), 1.22 (d, *J* = 15.0 Hz, 6H), 1.11 (t, *J* = 12.3 Hz, 2H). ^{13}C NMR (101 MHz, DMSO-d_6, 298K) δ 171.26, 156.56, 156.32, 137.77, 137.60, 128.82, 128.22, 128.18, 128.12, 65.76, 65.58, 55.04, 47.90, 32.86, 32.76, 32.41, 29.58, 25.69, 25.00, 24.95, 23.21. HR-ESI-MS (m/z): $C_{28}H_{37}N_3O_5$ $[M+H]^+$ calcd: 496.2811, found: 496.2811; $[M+Na]^+$ calcd: 518.2631, found: 518.2630.

Lys-OUA-Bn (A6)

white amorphous powder; Yield: 90 %; ^1H NMR (400 MHz, DMSO-d_6, 298K) was as follows: δ 8.39 (t, 1H), 7.39-7.29(m, 15H), 7.24 (m, 1H), 7.22 (m, 1H), 5.01 (d, *J* = 12.1 Hz, 4H), 4.27 (d, *J* = 5.6 Hz, 2H), 3.98 (m, *J* = 13.1, 8.3 Hz, 1H), 2.97-2.93 (m, 2H), 1.62-1.53 (m, 2H), 1.39-1.36 (m, 2H), 1.29-1.24 (m, *J* = 15.7, 7.5 Hz, 2H). ^{13}C NMR (101 MHz, DMSO-d_6, 298K) δ 172.49, 156.56, 156.50, 139.90, 137.76, 137.53,

128.82, 128.70, 128.20, 127.54, 127.18, 65.88, 65.60, 55.27, 42.49, 32.06, 29.54, 23.33. HR-ESI-MS (m/z): $C_{29}H_{33}N_3O_5$ $[M+H]^+$ calcd: 504.2498, found: 504.2505.

Lys-OUA-4-MeOPh (A7)

white amorphous powder; Yield: 92.3 %; ^{1}H NMR (400 MHz, DMSO-d_6 298K) was as follows: δ 9.88 (s, 1H), 7.51 (d, J = 8.8 Hz, 2H), 7.37-7.34 (m, 10H), 7.33 (s, 1H), 7.26 – 7.24 (m, 1H), 6.89 (d, J = 9.0 Hz, 2H), 5.02 (d, J = 15.7 Hz, 4H), 4.10 (q, J = 8.5 Hz, 1H), 3.73 (s, 3H), 2.99 (d, J = 5.6 Hz, 2H), 1.64 (m, J = 13.8, 10.3 Hz, 2H), 1.43-1.42 (m, J = 3.6 Hz, 2H), 1.37-1.25 (m, 2H). ^{13}C NMR (101 MHz, DMSO-d_6, 298K) δ 171.04, 156.58, 156.54, 155.70, 137.73, 137.49, 132.57, 128.82, 128.27, 128.18, 121.26, 114.30, 65.89, 65.60, 55.81, 55.64, 32.08, 29.56, 23.35. HR-ESI-MS (m/z): $C_{29}H_{33}N_3O_5$ $[M+H]^+$ calcd: 520.2448, found: 520.2446; $[M+Na]^+$ calcd: 542.2267, found: 542.2265.

Lys$_2$-PDA-BOC (A8)

white amorphous powder; Yield: 92.3 %; ^{1}H NMR (400 MHz, DMSO-d_6, 298K) was as follows: δ 7.88 (s, 2H), 7.37-7.34 (m, 20H), 7.31 (m, 2H), 7.25-7.22 (t, 2H), 5.02 (d, J = 5.7 Hz, 8H), 3.91 (m, J = 13.1, 8.0 Hz, 2H), 3.06-3.04 (m, 4H), 3.00-2.96 (m, 4H), 1.61-1.59 (m, 2H), 1.53-1.51 (d, J = 6.9 Hz, 4H), 1.44-1.36 (m, 4H), 1.30-1.26 (m, 4H). ^{13}C NMR (101 MHz, DMSO-d_6, 298K) δ 172.38, 156.56, 156.46, 137.75, 137.51, 128.82, 128.80, 128.19, 65.88, 65.59, 55.32, 36.59, 32.07, 29.66, 29.56, 23.33. HR-ESI-MS (m/z): $C_{47}H_{58}N_6O_{10}$ $[M+H]^+$ calcd: 867.4293, found: 867.4291.

Table S1. The gelation properties of **Lys-OUAs** in various solvents and fuel oils.

Solvent	A1	A2	A3	A4	A5	A6	A7	A8
Aniline	S	S	S	S	S	S	S	S
N,N-Dimethylaniline	S	S	S	S	G	S	S	G
Benzene	S	S	G	G	G	G	G	Is
Toluene	G	G	G	G	S	S	G	Is
Xylene	S	S	S	S	S	S	S	G
Phenylhydrazine	G	G	S	S	S	S	S	S
Chlorobenzene	S	S	S	S	G	S	G	G

Bromobenzene	S	S	S	G	G	S	G	G
Nitrobenzene	S	S	G	S	S	S	S	S
Ethylbenzene	G	G	G	G	G	S	G	G
Anisole	S	S	S	S	G	S	S	G
Acetone	S	Is	S	S	S	S	S	Is
Acetonitrile	S	G	PG	S	S	S	S	S
N,N-Dimethylformamide	S	S	S	S	S	S	S	S
Dimethyl sulfoxide	S	S	S	S	S	S	S	S
THF	S	S	S	S	S	S	S	S
1-Bromobutane	S	G	G	G	S	S	G	Is
Carbon tetrachloride	S	G	G	G	G	S	G	Is
Chloroform	S	S	S	S	S	S	S	S
n-Hexane	G	S	S	Is	S	Is	Is	Is
n-Heptane	G	G	S	Is	S	Is	Is	Is
Diesel	G	G	G	Is	S	Is	Is	Is
Crude oil	G	G	G	G	G	G	G	G
Petroleum ether	G	G	S	Is	S	Is	Is	Is
Gasoline	G	S	S	S	S	S	S	Is

G = gel state; PG = partial gel; S = solution state; Is = insoluble state. All the resulting gels could be stable at room temperature for several weeks.

Table S2. The critical gelation concentration (CGC) formed by **Lys-OUAs** in various solvents.

**Solvent**	**CGC/wt%**							
	**A1**	**A2**	**A3**	**A4**	**A5**	**A6**	**A7**	**A8**
Aniline	-	-	-	-	-	-	-	-
N,N-Dimethylaniline	-	-	-	-	4.95	-	-	0.69
Benzene	-	-	4.36	3.66	3.66	5.40	5.40	-
Toluene	5.43	3.69	2.49	3.18	-	-	5.43	-
Xylene	-	-	-	-	-	-	-	2.76

Solvent	CGC/wt%							
	A1	A2	A3	A4	A5	A6	A7	A8
Phenylhydrazine	4.35	2.22	-	-	-	-	-	-
Chlorobenzene	-	-	-	-	4.31	-	4.31	0.6
Bromobenzene	-	-	-	2.88	3.23	-	3.23	0.83
Nitrobenzene	-	-	2.03	-	-	-	-	-
Ethylbenzene	5.43	1.62	1.33	1.08	5.43	-	5.43	3.69
Anisole	-	-	-	-	3.24	-	-	1.65
Acetone	-	-	-	-	-	-	-	-
Acetonitrile	-	5.95	4.82	-	-	-	-	-
N,N-Dimethylformamide	-	-	-	-	-	-	-	-
Dimethyl sulfoxide	-	-	-	-	-	-	-	-
THF	-	-	-	-	-	-	-	-
1-Bromobutane	-	1.29	1.41	0.97	-	-	3.77	-
Carbon tetrachloride	-	5.9	0.69	0.52	2.05	-	3.04	-
Chloroform	-	-	-	-	-	-	-	-
n-Hexane	3.46	0.95	-	-	-	-	-	-
n-Heptane	3.85	2.86	-	-	-	-	-	-
Diesel	1.59	2.34	3.57	-	-	-	-	-
Crude oil	0.34	0.78	0.78	0.78	0.78	0.78	0.78	0.78
Petroleum ether	3.31	3.88	-	-	-	-	-	-
Gasoline	3.45	-	-	-	-	-	-	-

Table S3. The gelation properties of **Lys-OUA-C14** in various solvents and fuel oils

Solvent	direct addition	heating-cooling
Aniline	Is	S
N,N-Dimethylaniline	Is	S
Benzene	S	S
Toluene	G	G

Xylene	S	S
Phenylhydrazine	Is	G
Chlorobenzene	Is	S
Bromobenzene	Is	S
Nitrobenzene	Is	S
Ethylbenzene	G	G
Anisole	Is	S
Acetone	Is	S
Acetonitrile	S	S
N,N-Dimethylformamide	S	S
Dimethyl sulfoxide	S	S
THF	S	S
1-Bromobutane	S	S
Carbon tetrachloride	Is	S
Chloroform	S	S
n-Hexane	G	G
n-Heptane	G	G
Diesel	G	G
Gasoline	G	G
Kerosene	G	G
n-Dodecane	G	G
n-Tetradecane	G	G

Table S4. The critical gelation concentration (CGC), biphasic critical gelation concentration (BCGC) and phase transition temperature (T_{gel}) of the gels formed by **Lys-OUA-C14** in various solvents.

**Solvent**	**CGC/BCGC (wt%)**	T_{gel}
Toluene	5.43/5.82	43
Phenylhydrazine	4.35/4.95	46

Ethylbenzene	5.43/6.2	42
Diesel	1.59/2.38	63
Crude oil	0.34/2.54	73
Gasoline	3.45/4.5	44
Kerosene	2.04/2.52	68
n-Dodecane	2.6/3.2	63
n-Tetradecane	2.56/3.1	65
n-Hexane	3.46/4.2	64
n-Heptane	3.85/4.56	58
Petroleum ether	3.31/4.18	43

Table S5. A comparison of different materials for oil spill treatments.

**Comparative Study**						
**Reference [1-16]**	**Condition used**	**CGC/MGC**	**Biphasic CGC/MGC**	**Oil removal efficiency (%)**	**Time for solidifying oil**	**Reusability of gelators**
Dipeptide-based amphiphilic gelator, ***RSC Advances*** (Ravishankar et al., 2016)	Carrier solvent (toluene)	1 % w/v for diesel and 2 % w/v for crude oil (CGC)	1.2 % w/v for diesel and 2 % w/v for crude oil (BCGC)	95% diesel oil removal efficiency and 60-70 % crude oil removal efficiency	Few minutes	Unspecified
Sugar-based gelator, ***Angew Chem Int Ed*** (Vibhute et al., 2016)	Powder	0.5 % w/v for crude oil (MGC)	0.6 % w/v for crude oil (BMGC)	High efficiency (%value unspecified)	Unspecified	non-reusable
Sugar-derived oleogelator, ***Angew Chem Int Ed*** (Prathap et al., 2017)	Carrier Solid (cellulose pulp)	Unspecified	Unspecified	High efficiency (%value unspecified)	30minutes -2 hours	Unspecified
Fmoc-based gelator, ***Angew Chem Int Ed*** (Ren et al., 2017)	Wetting Solvent (CH_3CN)	2.4 % w/v for crude oil (MGC)	3.5 % w/v for crude oil (BMGC)	High efficiency (%value unspecified)	14 minutes	Unspecified

Sorbitol-based gelator, ***J Appl Polym Sci*** (Zhuan et al., 2018)	Powder	0.04 % w/v for crude oil (MGC)	0.04 % w/v for crude oil (BMGC)	80 % oil removal efficiency	17 minutes	Reuseable, 90 % of gelator recovered after 10 cycles
Tyrosine-based dipeptide gelator, ***ACS Omega*** (Majumder et al., 2019)	Carrier solvent (toluene and PE)	Unspecified	Unspecified	High efficiency (%value unspecified)	Unspecified	Unspecified
Oleylamide derivatives organogelators, ***J Hazard Mater*** (Eftekhari-Sis et al., 2019)	Powder	3.3 % w/v for crude oil (CGC)	Unspecified	High efficiency (%value unspecified)	5 minutes	Unspecified
Arabinose derivatives organogelators, ***Chem Commun*** (Pathak et al., 2020)	Powder	0.25 % w/v for crude oil (CGC)	Unspecified	High efficiency (%value unspecified)	12hours	Unspecified
10-undecenioc acidderivatives organogelators, ***Langmuir*** (Soares et al., 2022)	Powder	9.5 % w/v for diesel (CGC)	Unspecified	High efficiency (%value unspecified)	17 minutes	Unspecified
Amide-based organogelators, ***J Mater Chem A*** (Yang et al., 2023)	Powder (Heat)	0.75 % w/v for diesel (CGC)	Unspecified	High efficiency (%value unspecified)	Unspecified	Unspecified
Mandelic acid-derived organogelators, ***Langmuir*** (Zhang et al., 2024)	Powder	3.0 % w/v for crude oil (CGC)	6.0 % w/v for crude oil (BCGC)	90 % oil removal efficiency	30 seconds	Reusable
Dicholesteryl derivatives Organogelators,	Powder	1 % w/v for diesel (CGC)	Unspecified	High efficiency (%value	Unspecified	Reusable

Sci Rep (Googol et al., 2024)				unspecified)		
D-gluconic acetal-based organogelators, ***Langmuir*** (Wang et al., 2025)	Powder	1.87 % w/v for crude oil (CGC)	Unspecified	95.3 % oil removal efficiency	Unspecified	Reusable, 96.2 % of gelator recovered
Glutamine derivatives Organogelators, ***Adv Mater Interfaces*** (Zhang et al., 2025)	Carrier solvent (Ethanol)	Unspecified	Unspecified	78-85% diesel removal efficiency	150 seconds	Reusable
Benzimidazole-Based Organogelators, ***J Mol Struct*** (Zainab et al., 2025)	Powder	1.0 % w/v for diesel oil and 2.2 % w/v for crude oil (CGC)	1.6 % w/v for diesel oil and 3.0 % w/v for crude oil (BCGC)	95 % oil removal efficiency	5 minutes for diesel and 15-20 minutes for crude oil	Reuseable, 92 % of gelator recovered after 4 recovery cycles
Tripodal arylhydrazone based PSOG ***Chem Eng J*** (Pegu et al., 2026)	Powder (Heat)	1.5 mg/mL for diesel and 2 mg/mL for kerosene (CGC)	Unspecified	83.33 % oil removal efficiency	5 minutes	Unspecified
Lysine-derived organogelators, (Current Work)	Powder	1.59 wt% (1.35 % w/v) for diesel and 0.34 wt% (0.28 % w/v) for crude oil (CGC)	2.38 wt% (2.02 % w/v) for diesel oil and 2.54 wt% (2.18 % w/v) for crude oil (BCGC)	95.9% diesel oil removal efficiency and 96.3 % crude oil removal efficiency	120 seconds for diesel and 10 minutes for crude oil	Reusable, 95.2 % of gelator recovered

Table S6. Variation of gelator mass and diesel recovery rate with repeated cycles

Recovery Time	Lys-OUA-C14(mg)	Diesel removal rate(%)
1	28.28	95.90
2	26.92	93.65
3	26.16	92.59
4	25.23	91.35
5	24.61	90.60

Table S7. Variation of gelator mass and crude oil recovery rate with repeated cycles

Recovery Time	Lys-OUA-C14(mg)	Crude oil removal rate(%)
1	40	96.32
2	37.54	94.65
3	34.32	91.59
4	32.56	89.35
5	31.83	87.60

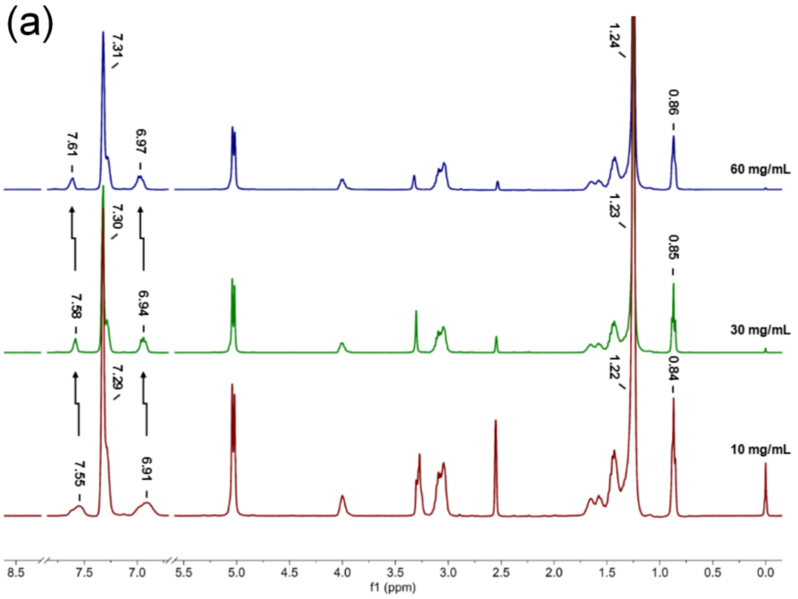

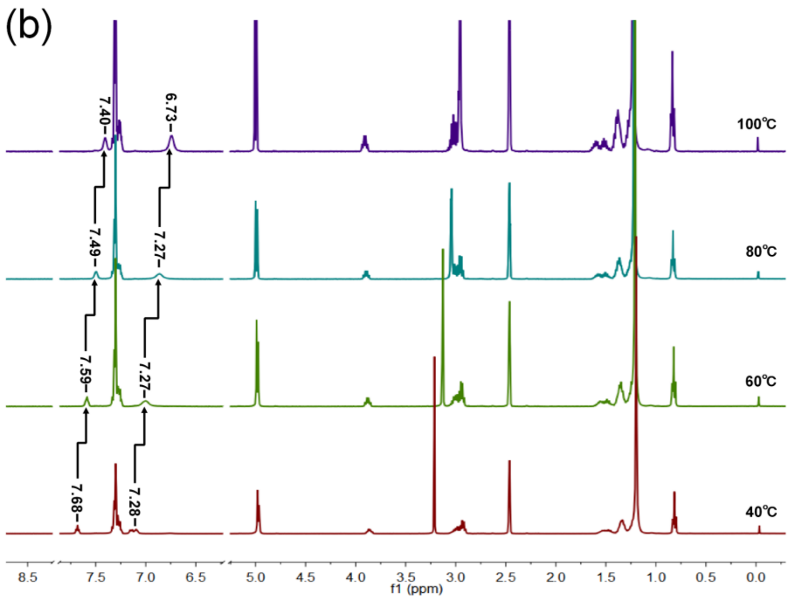

Figure S1. (a) Partial ^{1}H NMR spectra of **Lys-OUA-C14** in DMSO-d_6/$CDCl_3$ (v/v = 1:1) at various concentrations (400MHz, 298 K); (b) temperature-dependence ^{1}H NMR spectra of **Lys-OUA-C14** in DMSO-d_6 recorded upon heating from 40 °C to 100 °C

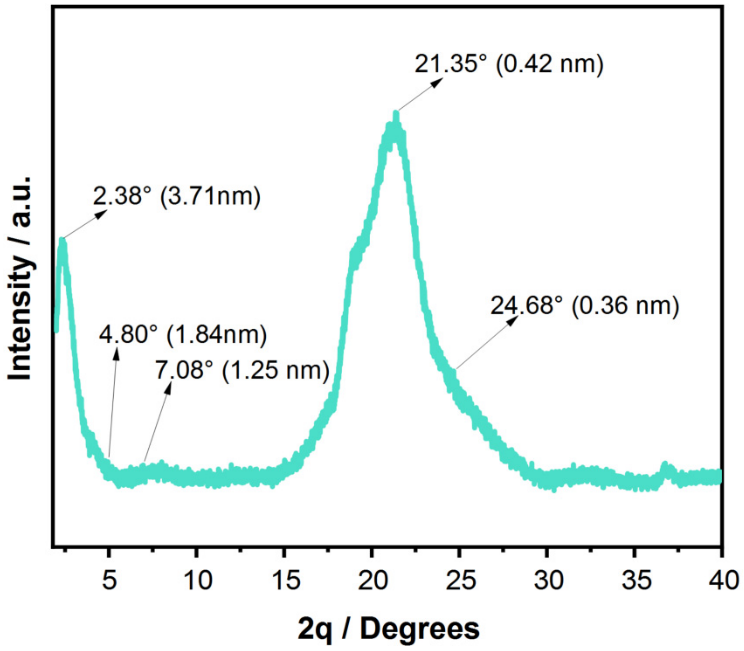

Figure S2. Powder X-ray diffraction pattern of **Lys-OUA-C14** in toluene xerogel.

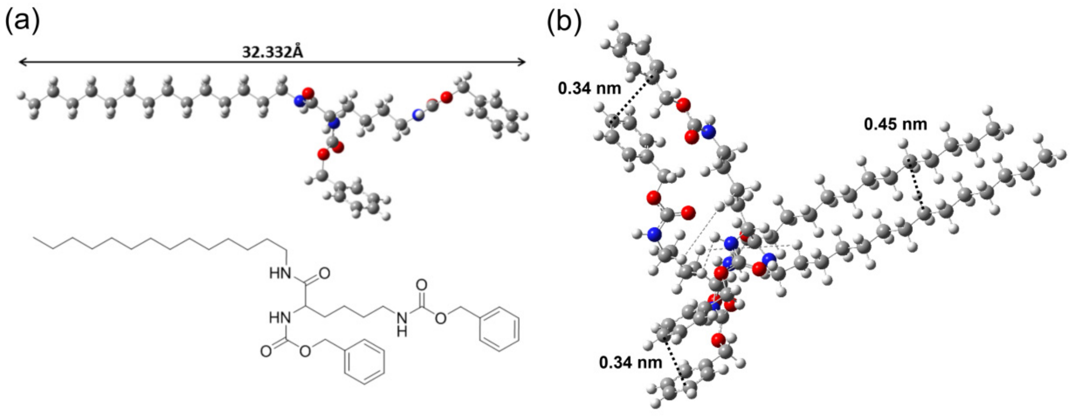

Figure S3. (a) The optimized structure of molecule **Lys-OUA-C14** calculated using method DFT//B3LYP/6-31G (d) as carried out by the Gaussian 09 package; (b) DFT calculations for intermolecular distances between two **Lys-OUA-C14** molecules.

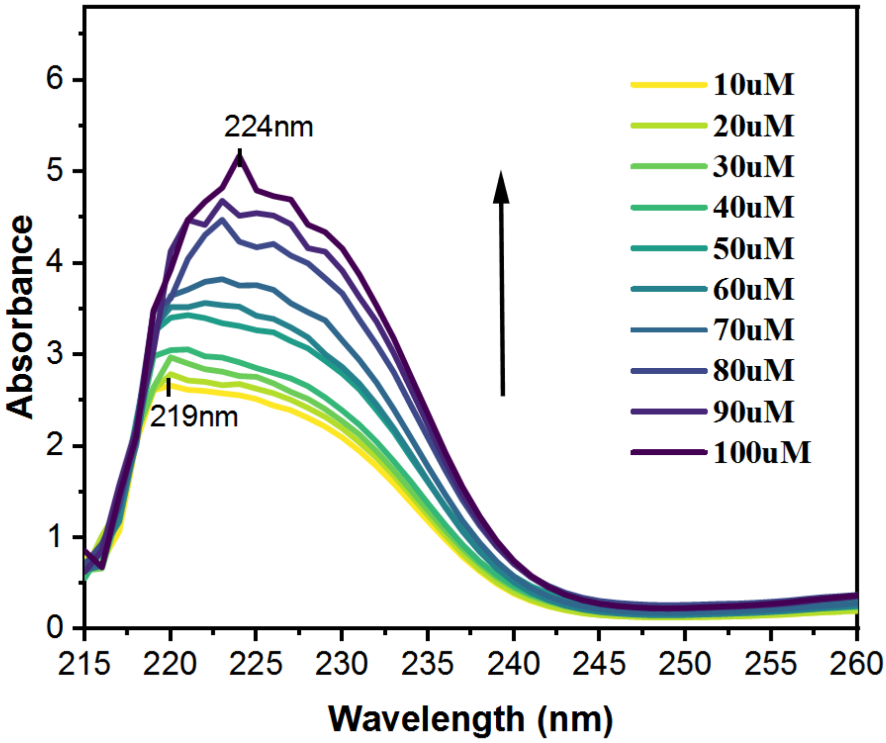

Figure S4. UV/vis absorption titration spectra of **Lys-OUA-C14** in *n*-heptane.

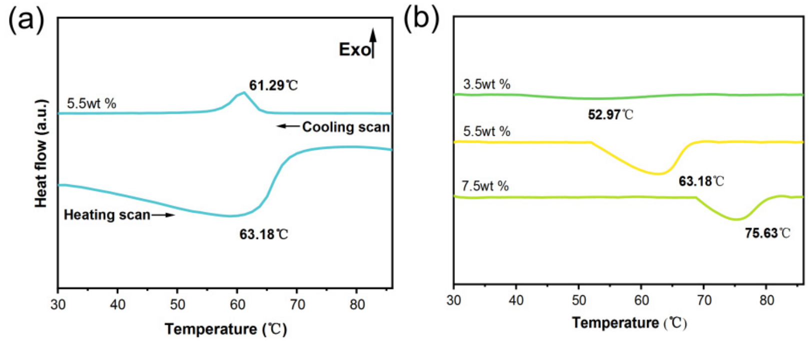

Figure S5. (a) DSC thermograms of the gels formed from **Lys-OUA-C14** in *n*-hexane (5.5 wt%); (b) Lys-OUA-C14 in *n*-hexane with different gelator concentrations (3.5 wt%, 5.5wt% and 7.5 wt%).

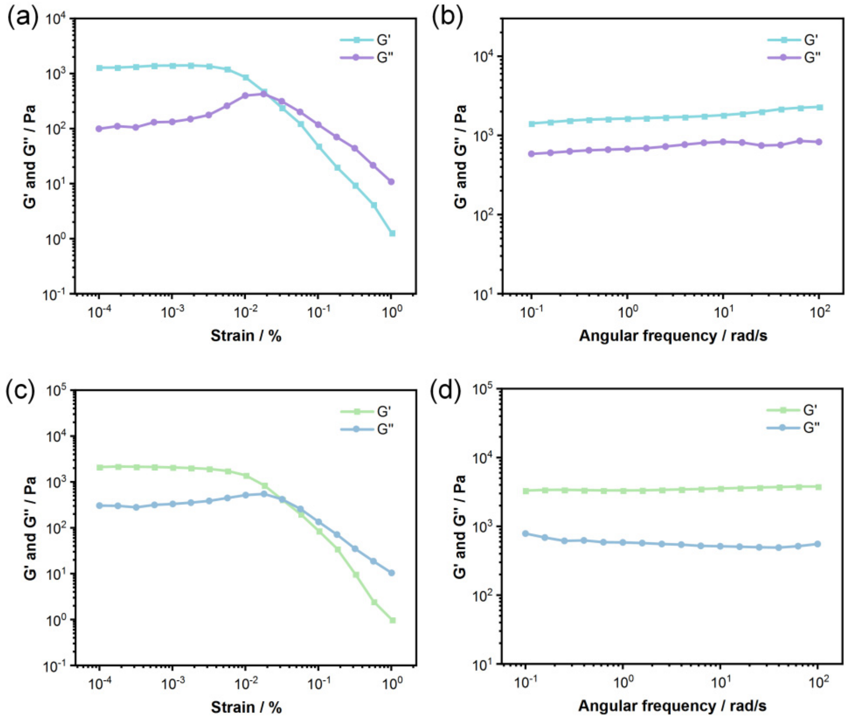

Figure S6. Rheological properties of **Lys-OUA-C14** (3.5 wt %) in different oil. (a) Stress sweep of petroleum ether organogels; (b) Frequency scan of petroleum ether organogels; (c) Stress sweep of *n*-hexane organogels; (d) Frequency scan of *n*-hexane organogels. G′ and G″ are storage and loss moduli, respectively.

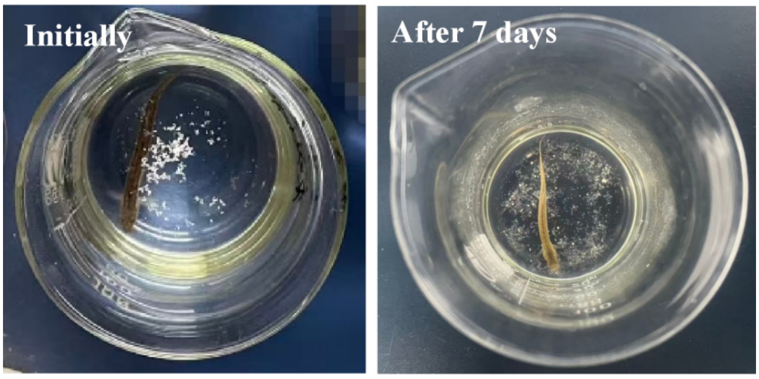

Figure S7. Biotoxicity test of gelator **Lys-OUA-C14** over 7 days.

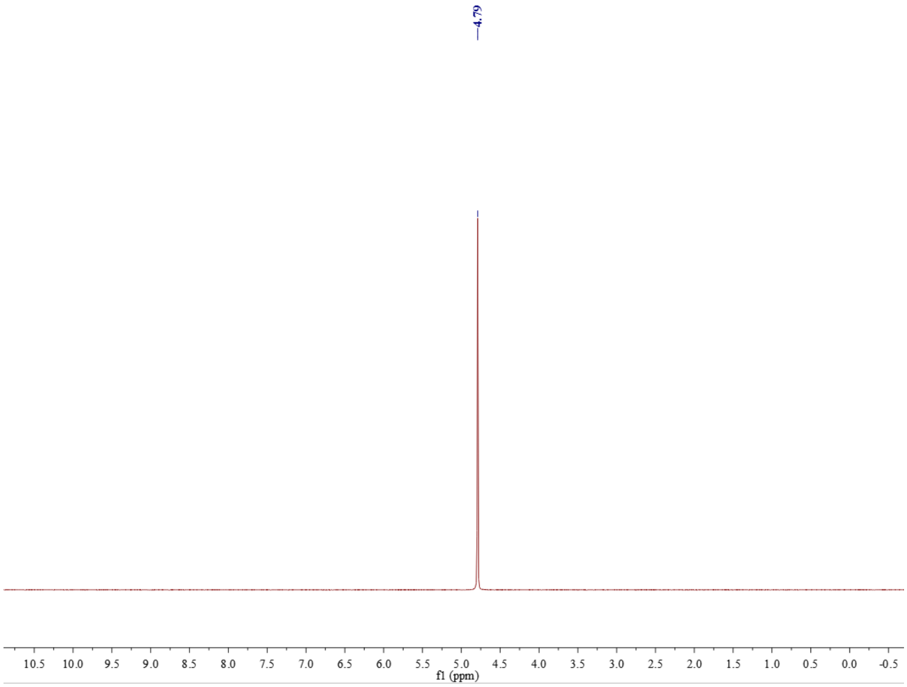

Figure S8. ^{1}H NMR spectrum (400 MHz, D_2O, 298 K) of **Lys-OUA-C14**.

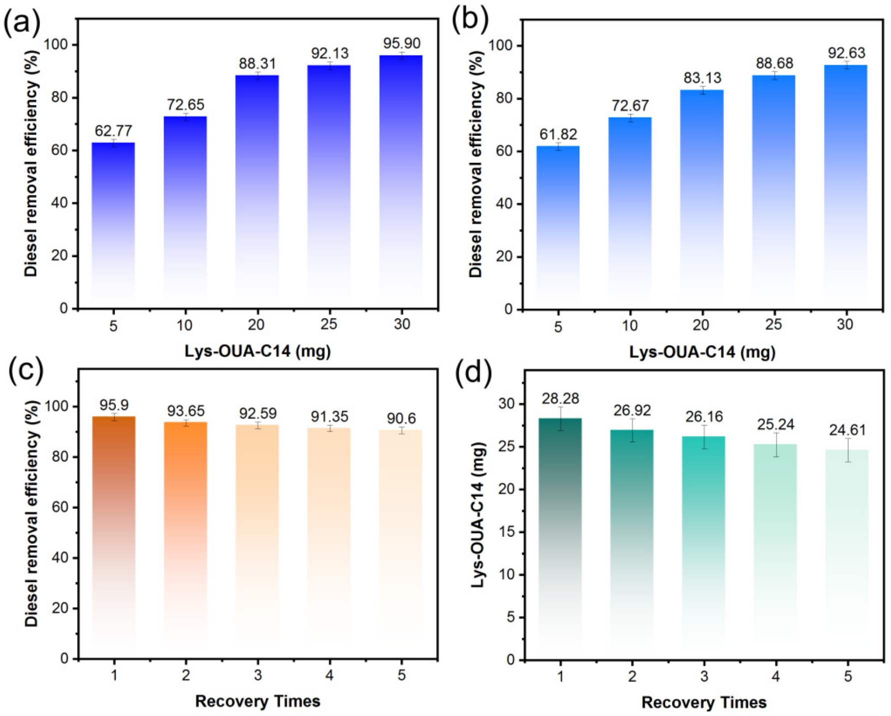

Figure S9. (a), (b) Diesel removal efficiency at different gelator dosages with diesel/seawater ratios of 1:10 and 1:20, respectively; (c) Diesel removal efficiency; (d) Recovery efficiency of **Lys-OUA-C14**. Data are mean values from triplicate experiments and error bars indicate SD.

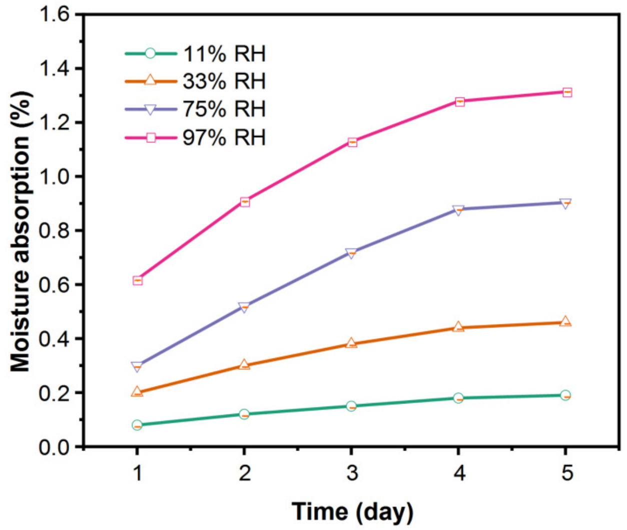

Figure S10. Moisture absorption of **Lys-OUA-C14** powder (10 mg) at 25℃ under different relative humidities over 5 days. Data are presented as mean ± SD (n = 3).

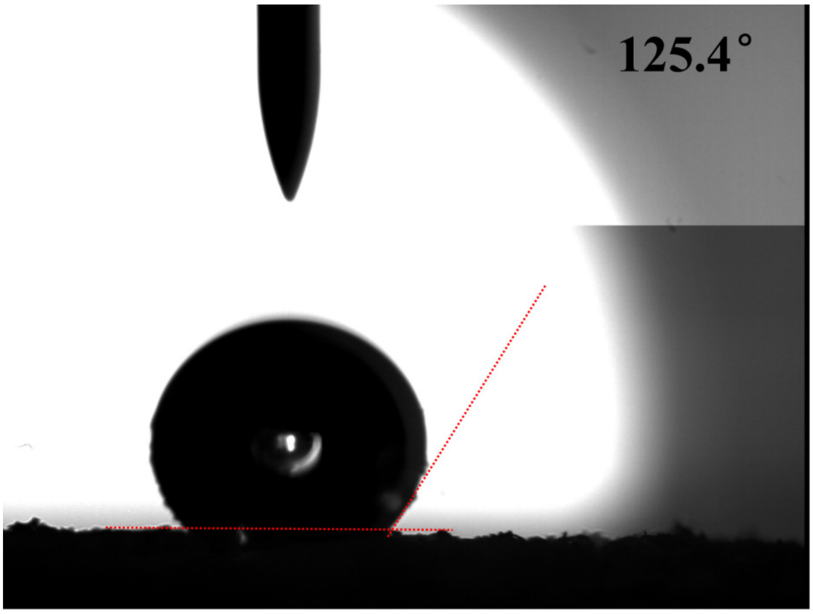

Figure S11. The contact angle of **Lys-OUA-C14** surface measured by the pendant drop method at room temperature.

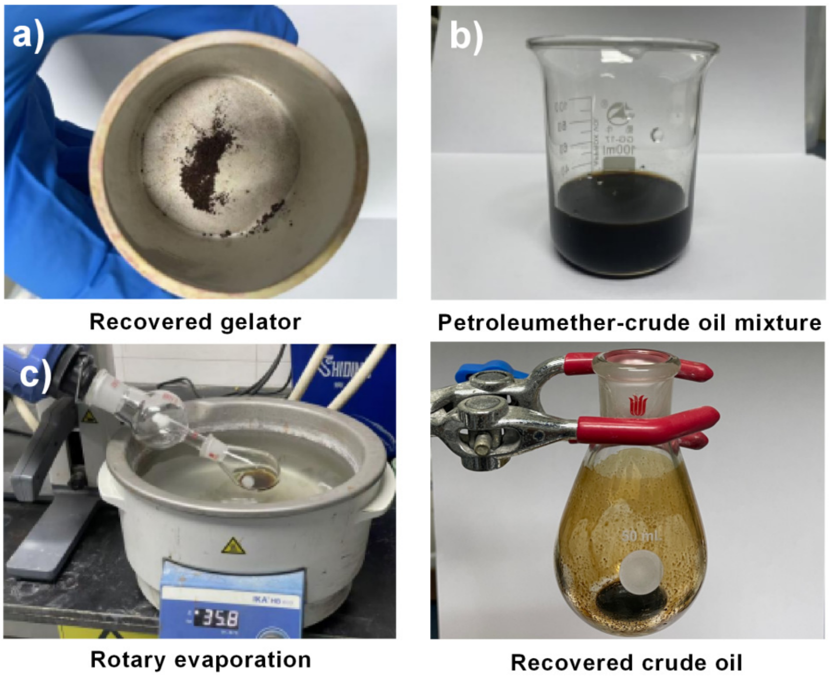

Figure S12. Demonstration of crude oil recovery from the gel formed with **Lys-OUA-C14** as the phase-selective organogelator.

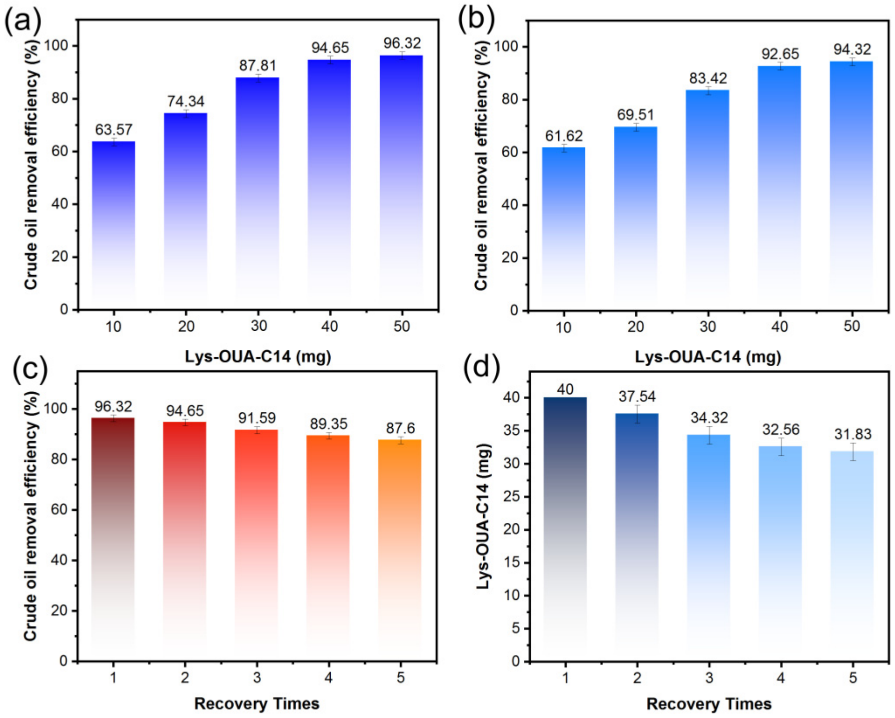

Figure S13. (a), (b) Crude oil removal efficiency at different gelator dosages with crude oil/seawater ratios of 1:10 and 1:20, respectively; (c) Crude oil removal efficiency; (d) Recovery efficiency of **Lys-OUA-C14**. Data are mean values from triplicate experiments and error bars indicate SD.

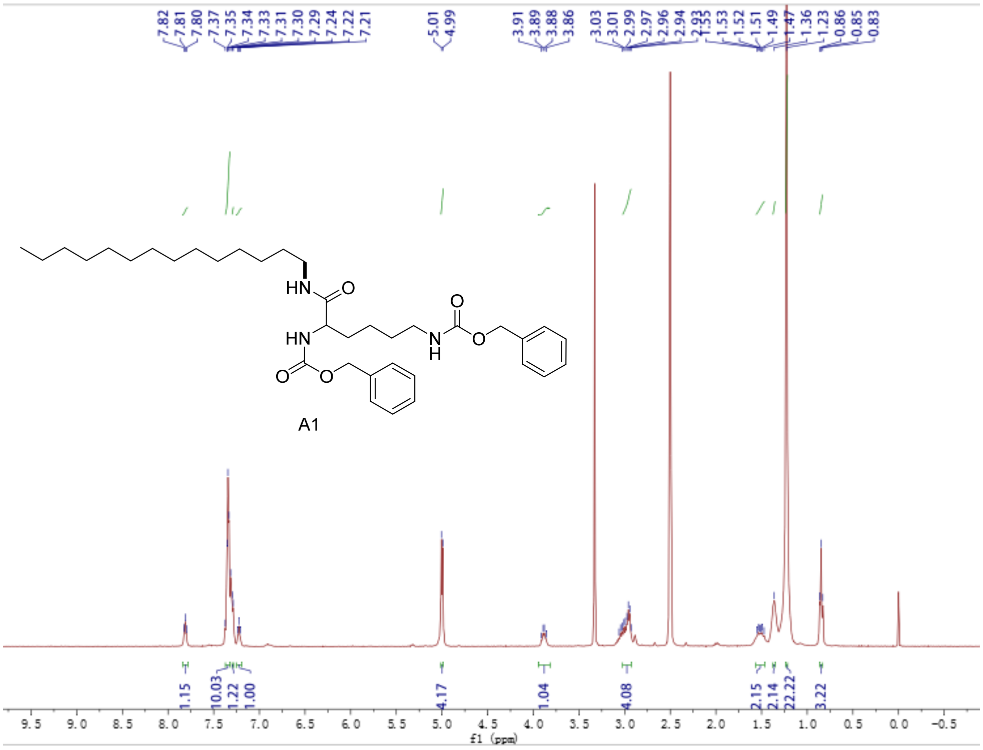

Figure S14. ^{1}H NMR spectrum (400 MHz, DMSO-d_6, 298K) of **A1**.

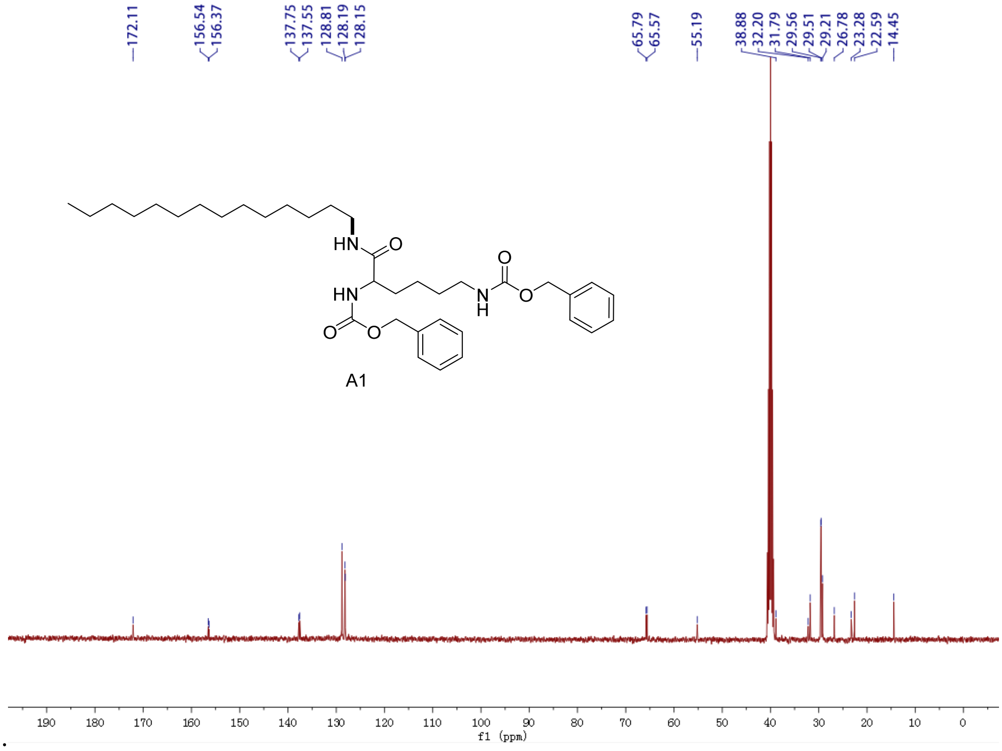

Figure S15. ^{13}C NMR spectrum (101 MHz, DMSO-d_6, 298K) of **A1**.

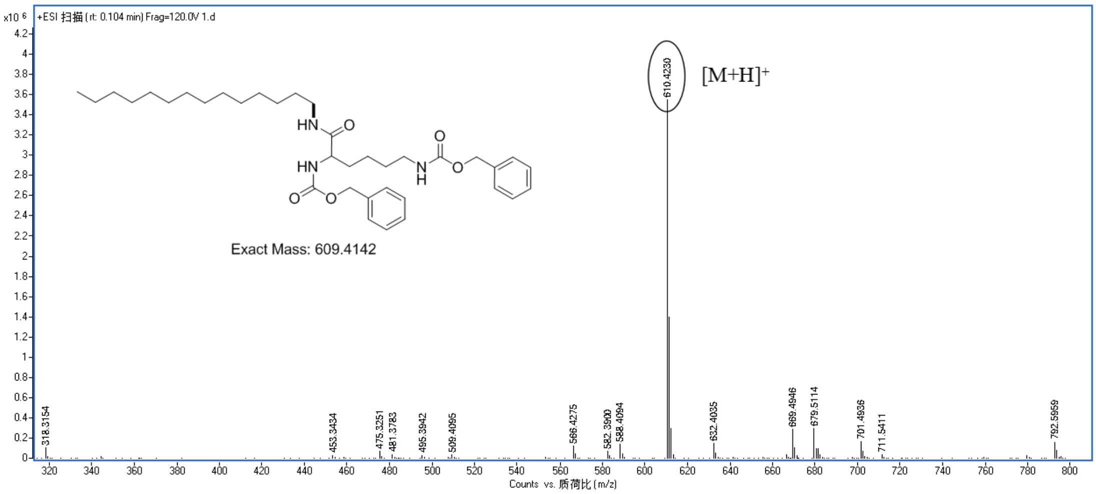

Figure S16. Mass spectrum of **A1**.

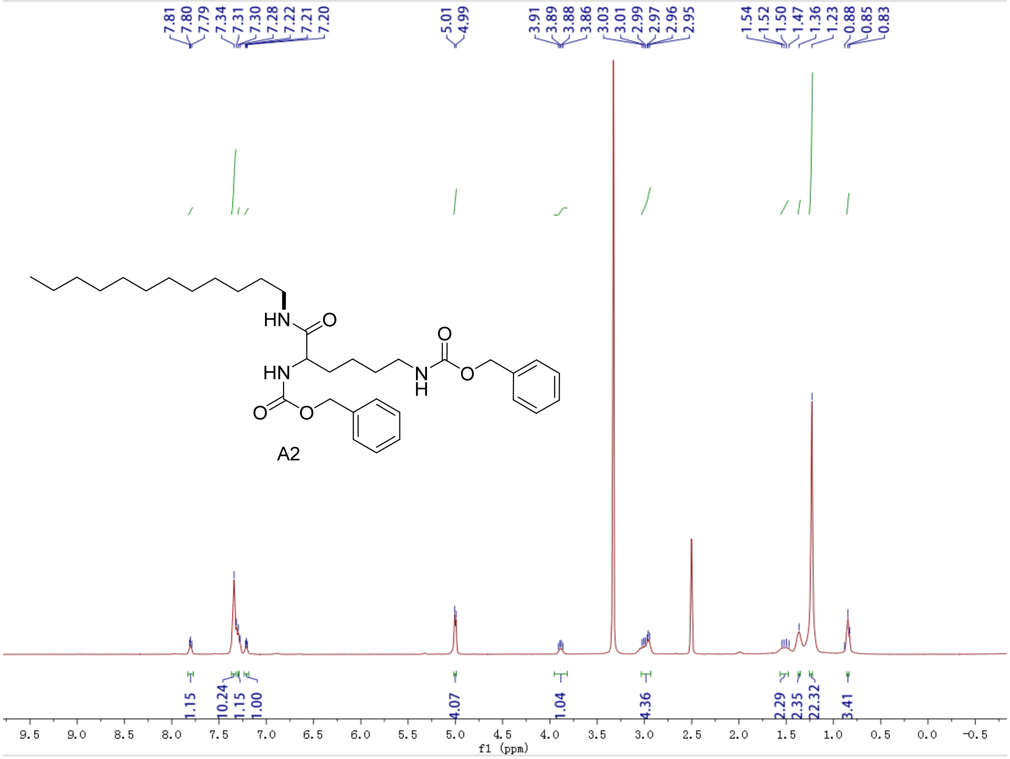

Figure S17. ^{1}H NMR spectrum (400 MHz, DMSO-d_6, 298K) of **A2**.

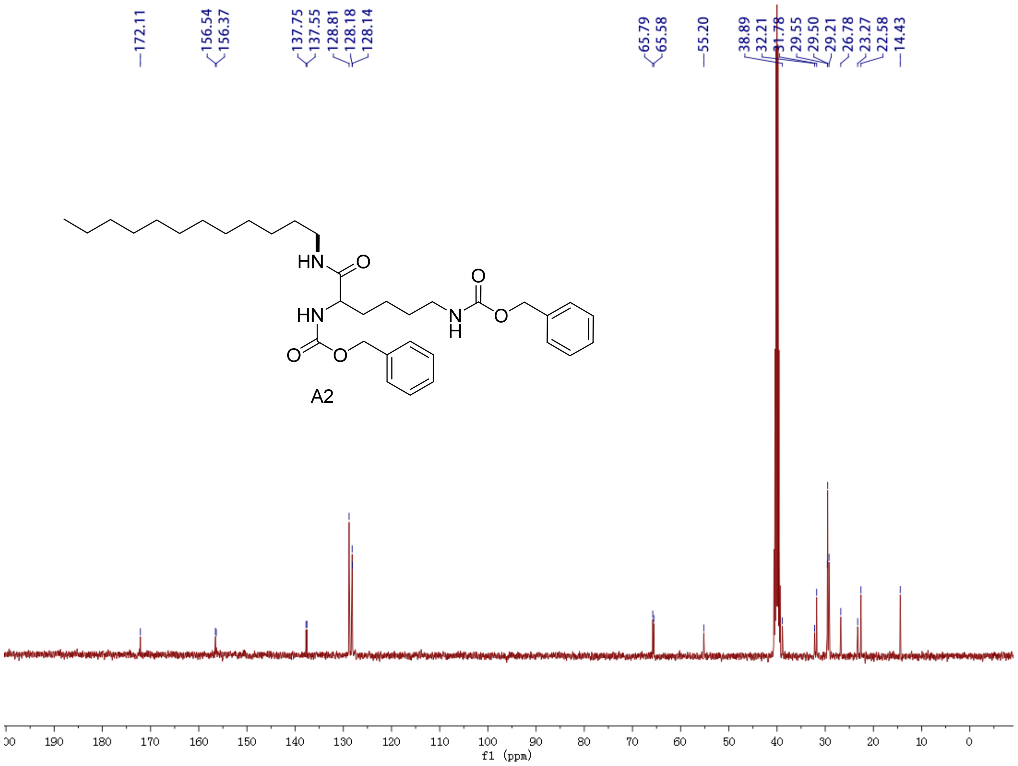

Figure S18. ^{13}C NMR spectrum (101 MHz, DMSO-d_6, 298K) of **A2**.

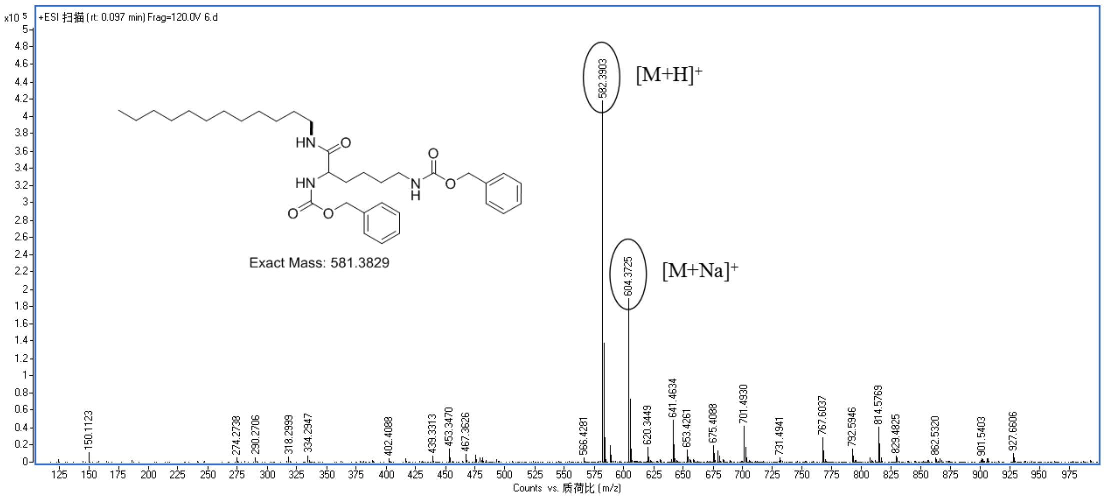

Figure S19. Mass spectrum of **A2**.

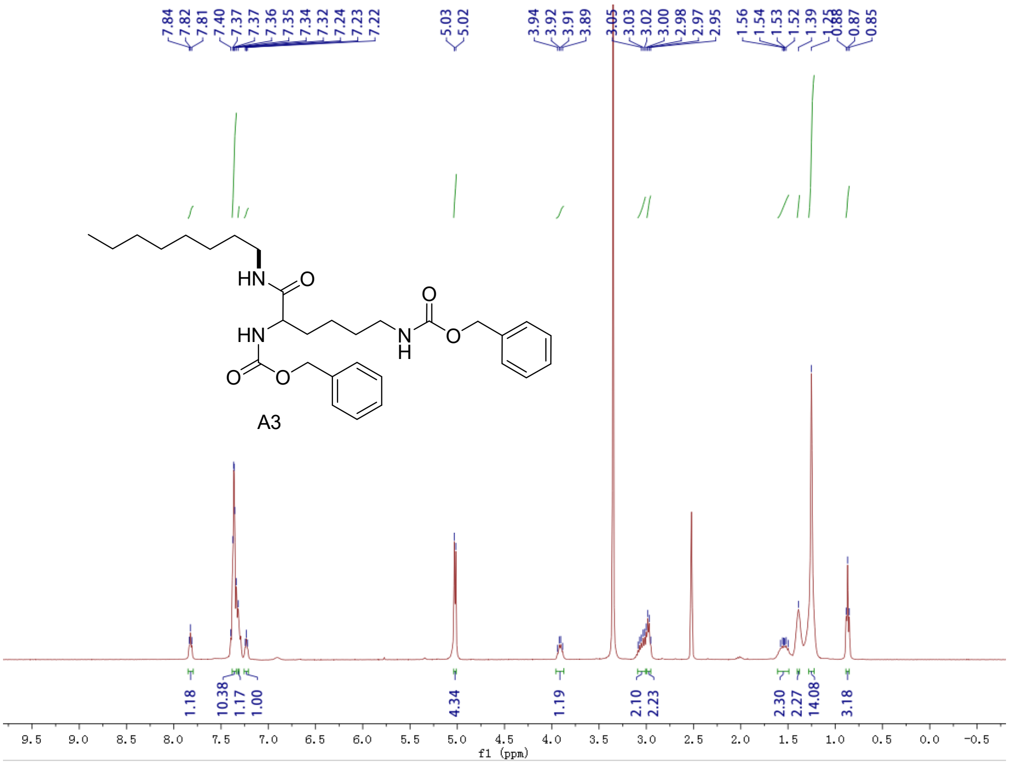

Figure S20. ^{1}H NMR spectrum (400 MHz, DMSO-d_6, 298K) of **A3**.

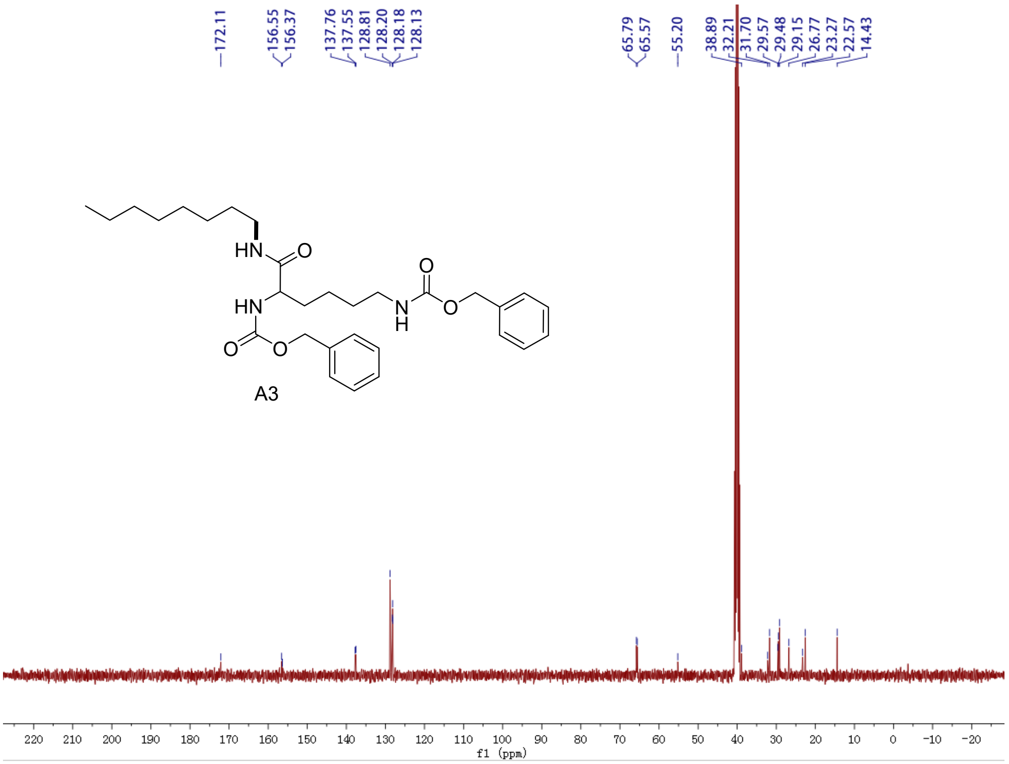

Figure S21. ^{13}C NMR spectrum (101 MHz, DMSO-d_6, 298K) of **A3**.

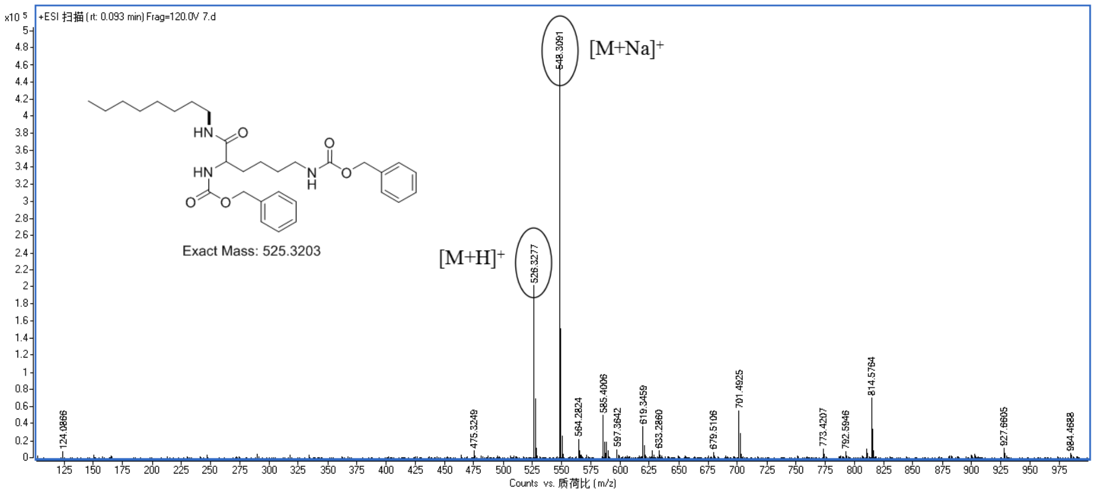

Figure S22. Mass spectrum of **A3**.

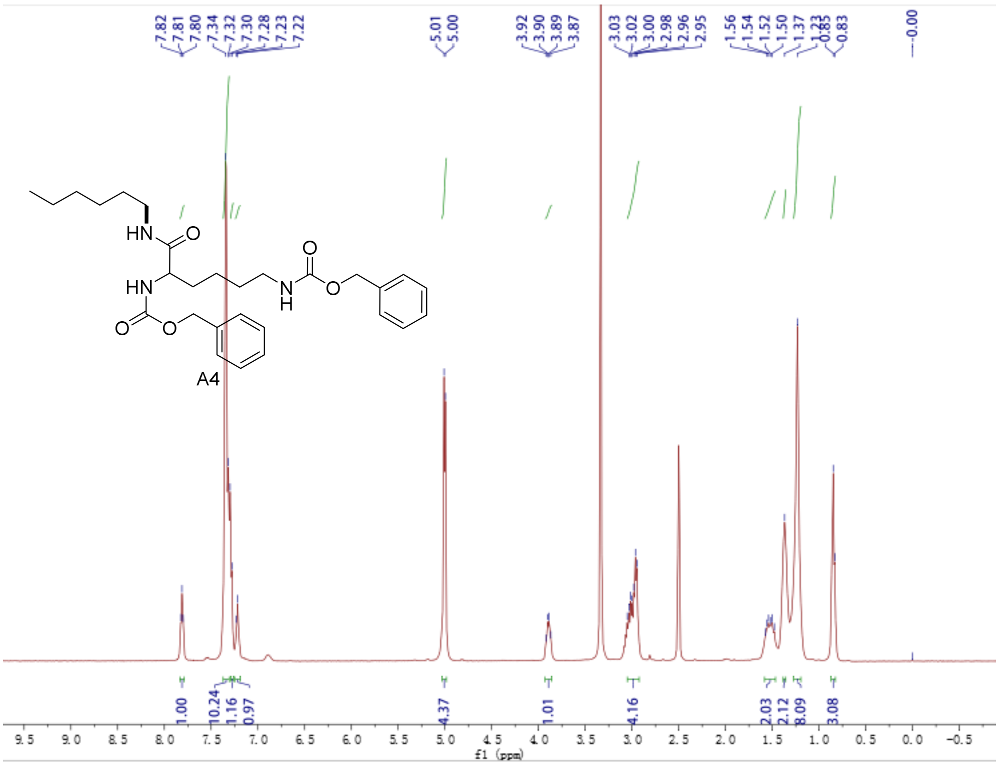

Figure S23. ^{1}H NMR spectrum (400 MHz, DMSO-d_6, 298K) of **A4**.

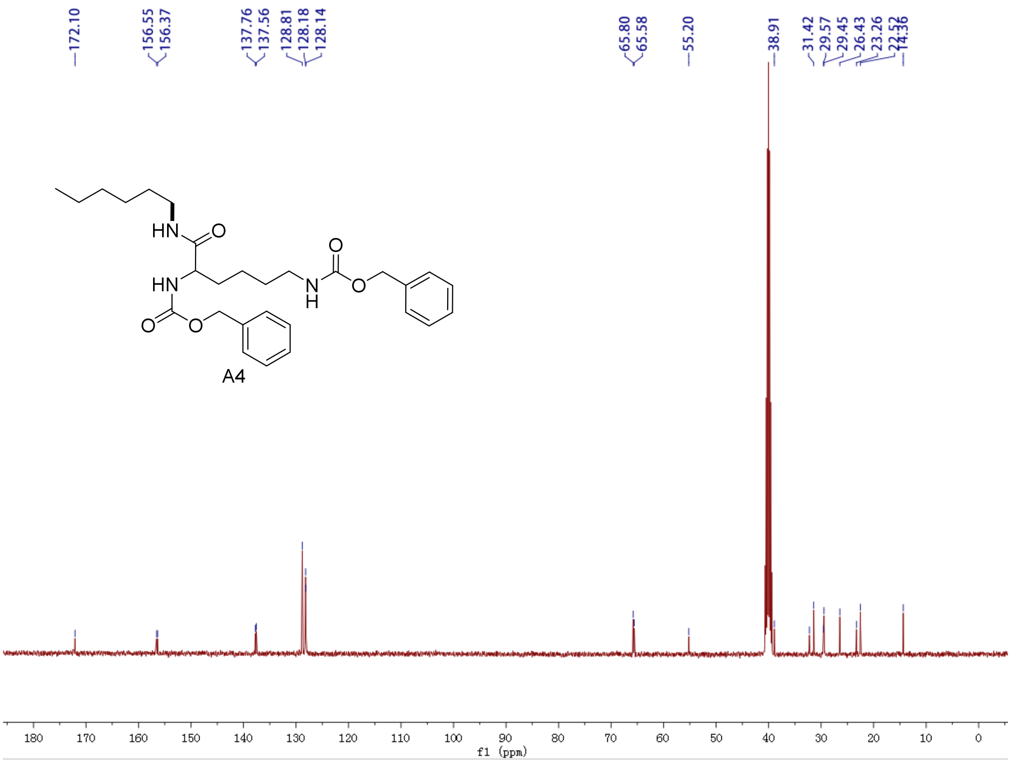

Figure S24. ^{13}C NMR spectrum (101 MHz, DMSO-d_6, 298K) of **A4**.

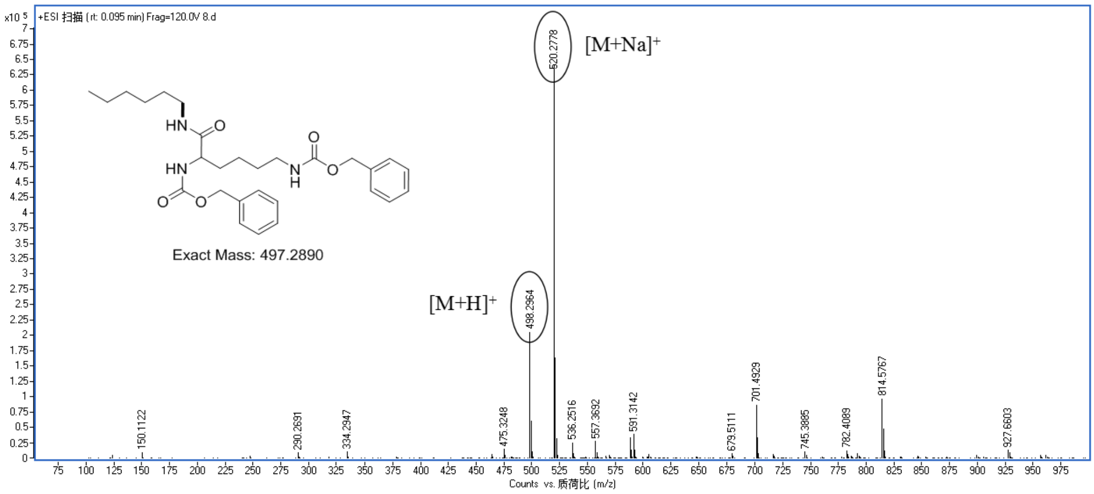

Figure S25. Mass spectrum of **A4**.

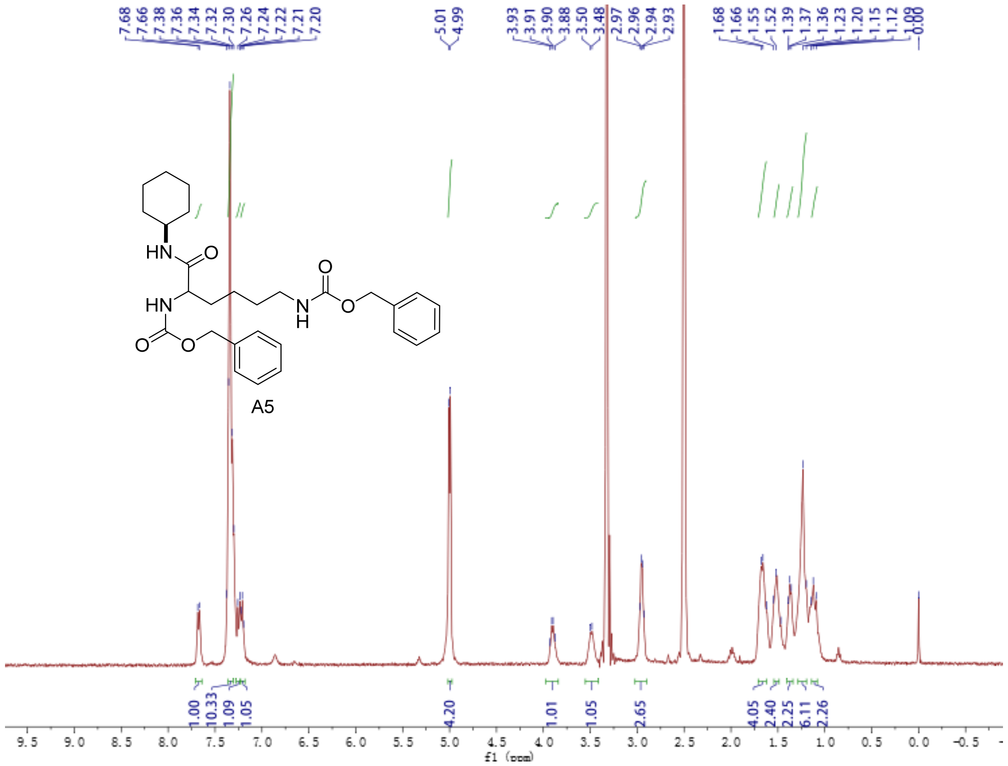

Figure S26.^{1}H NMR spectrum (400 MHz, DMSO-d_6, 298K) of **A5**.

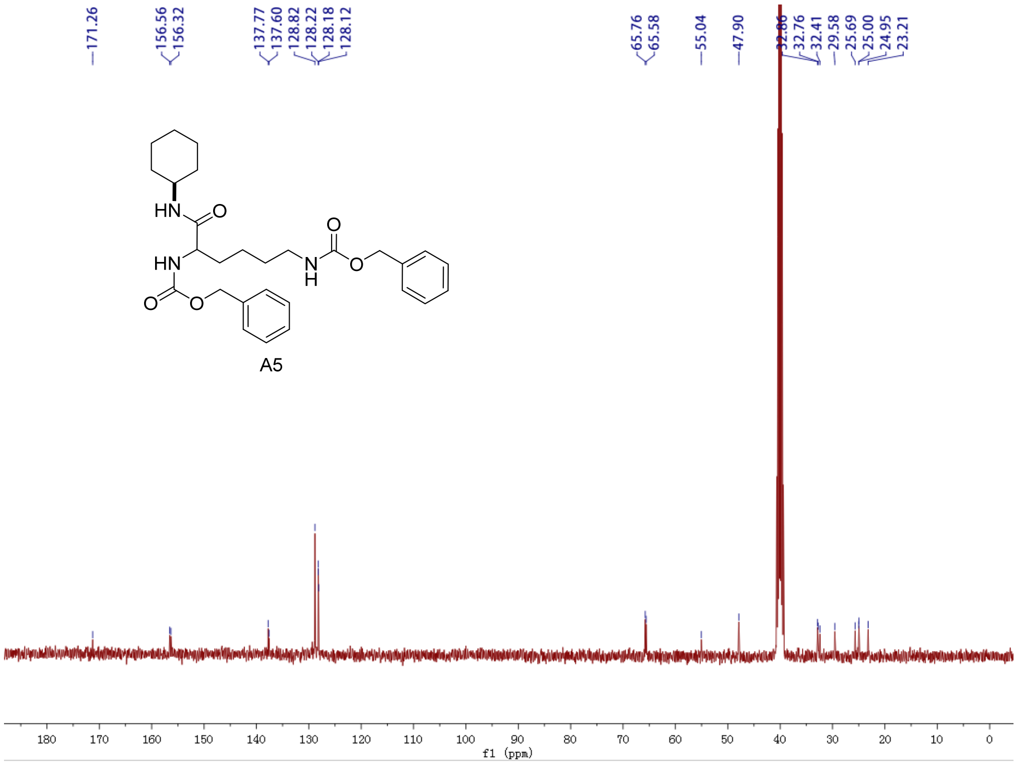

Figure S27. ^{13}C NMR spectrum (101 MHz, DMSO-d_6, 298K) of **A5**.

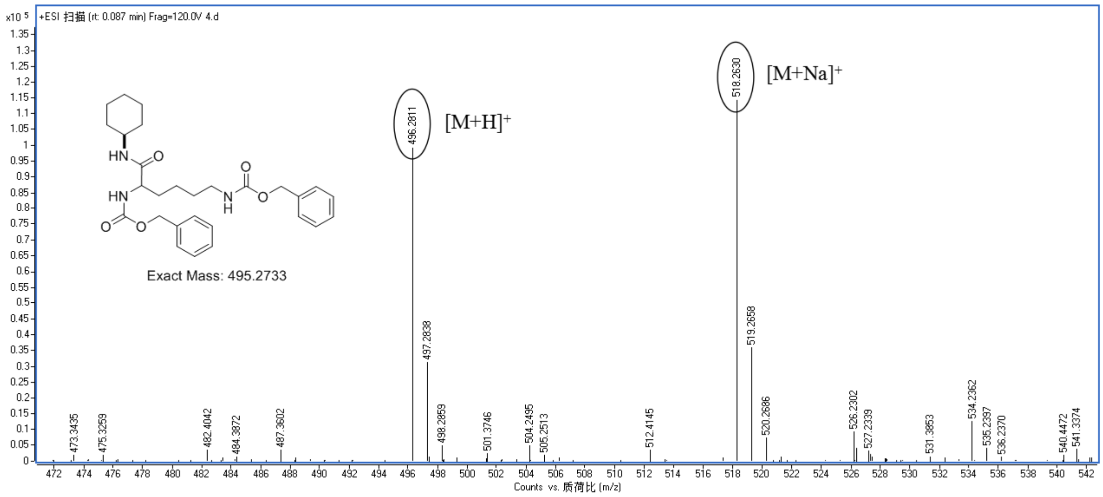

Figure S28. Mass spectrum of **A5**.

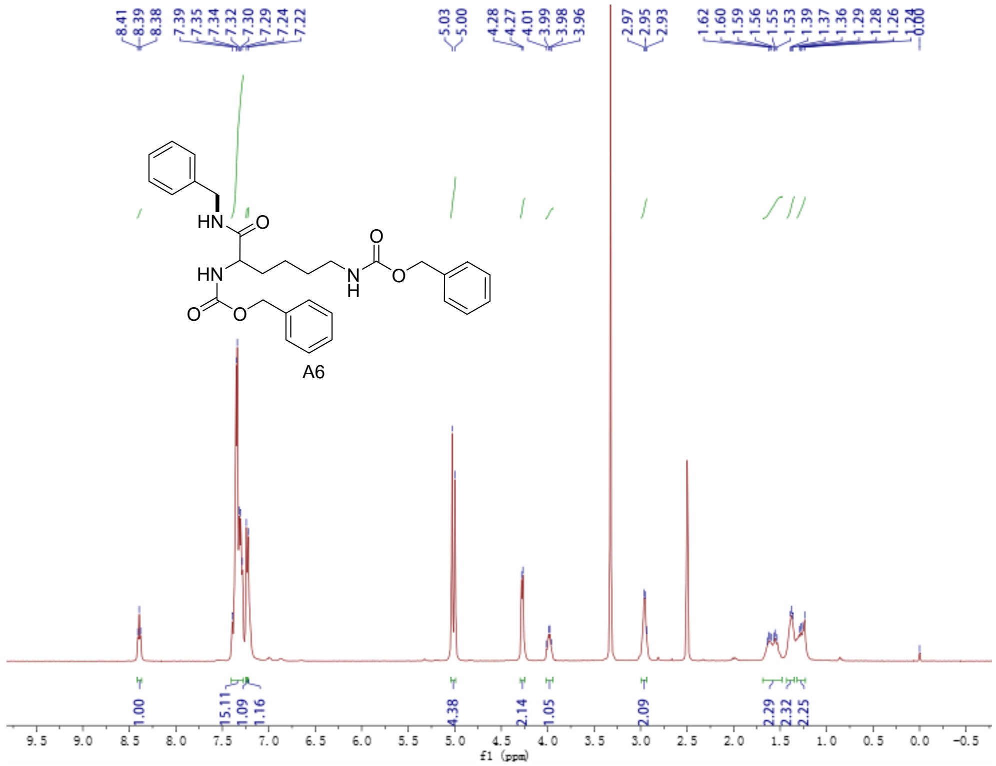

Figure S29. ^{1}H NMR spectrum (400 MHz, DMSO-d_6, 298K) of **A6**.

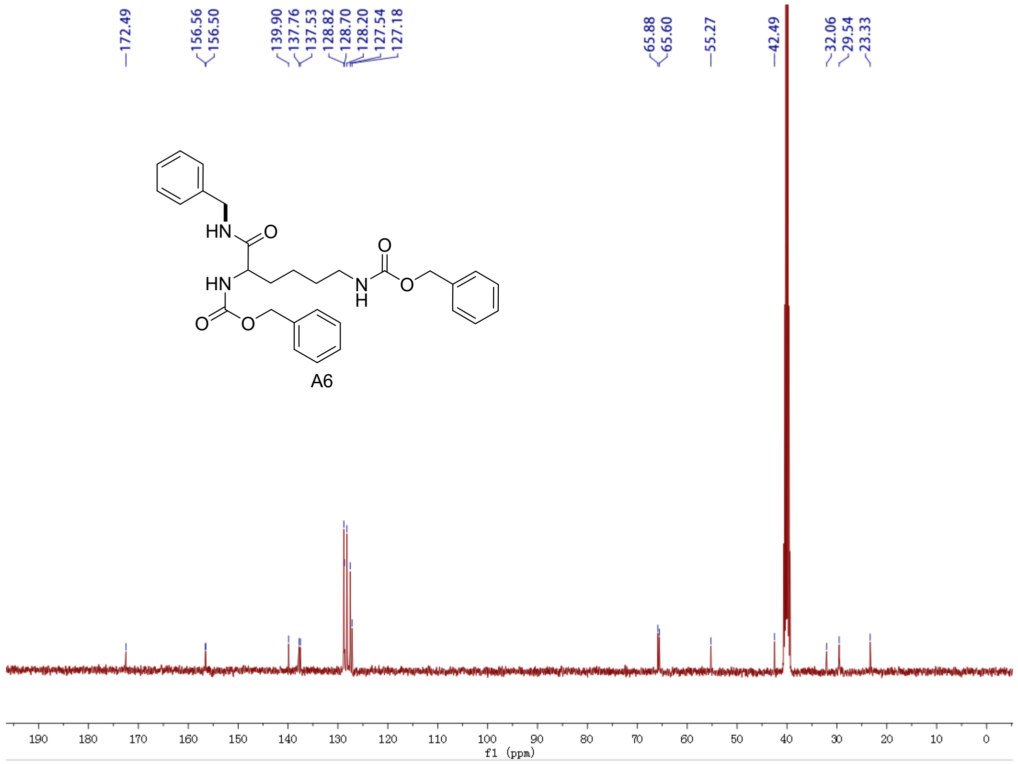

Figure S30. ^{13}C NMR spectrum (101 MHz, DMSO-d_6, 298K) of **A6**.

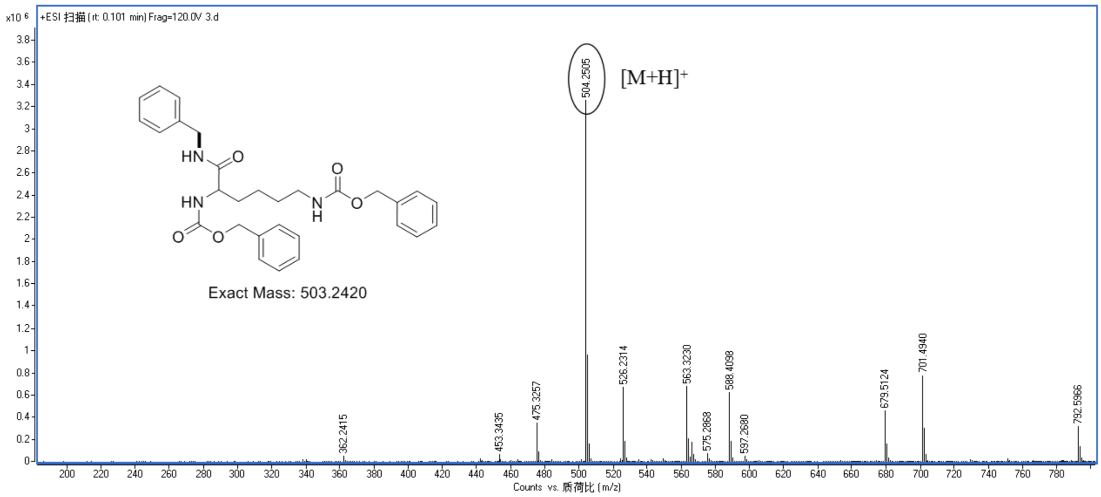

Figure S31. Mass spectrum of **A6**.

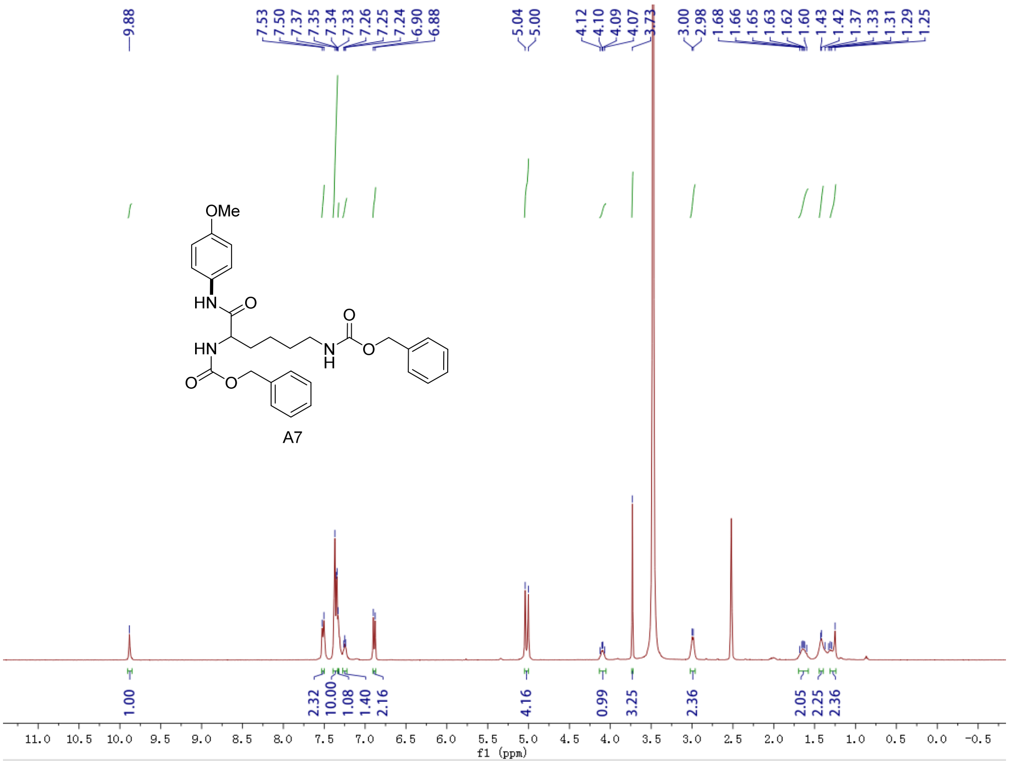

Figure S32. ^{1}H NMR spectrum (400 MHz, DMSO-d_6, 298K) of **A7**.

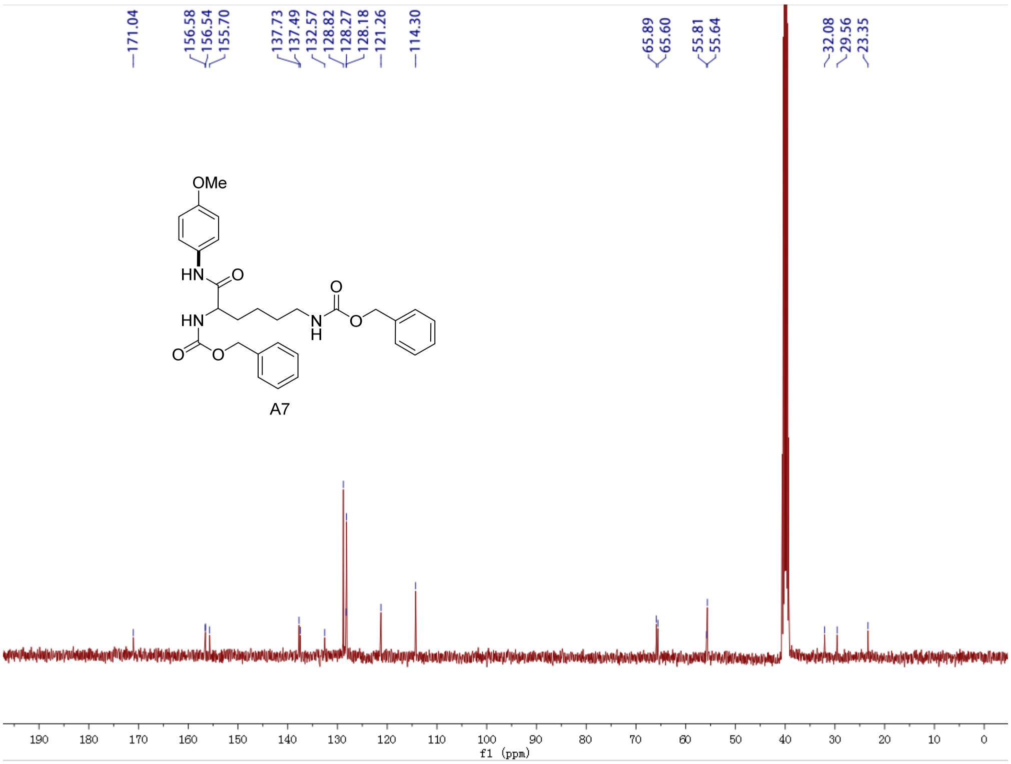

Figure S33. ^{13}C NMR spectrum (101 MHz,DMSO-d_6, 298K) of **A7**.

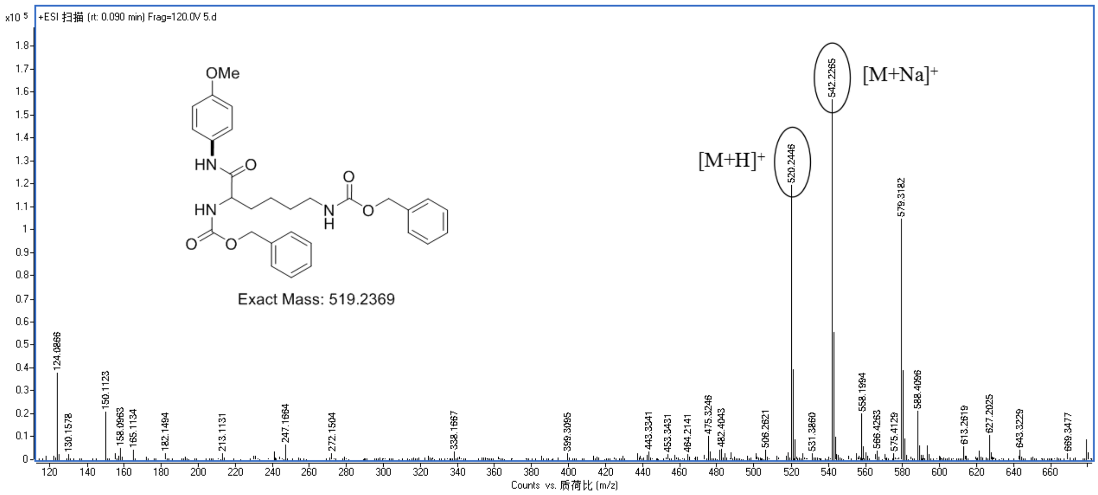

Figure S34. Mass spectrum of **A7**.

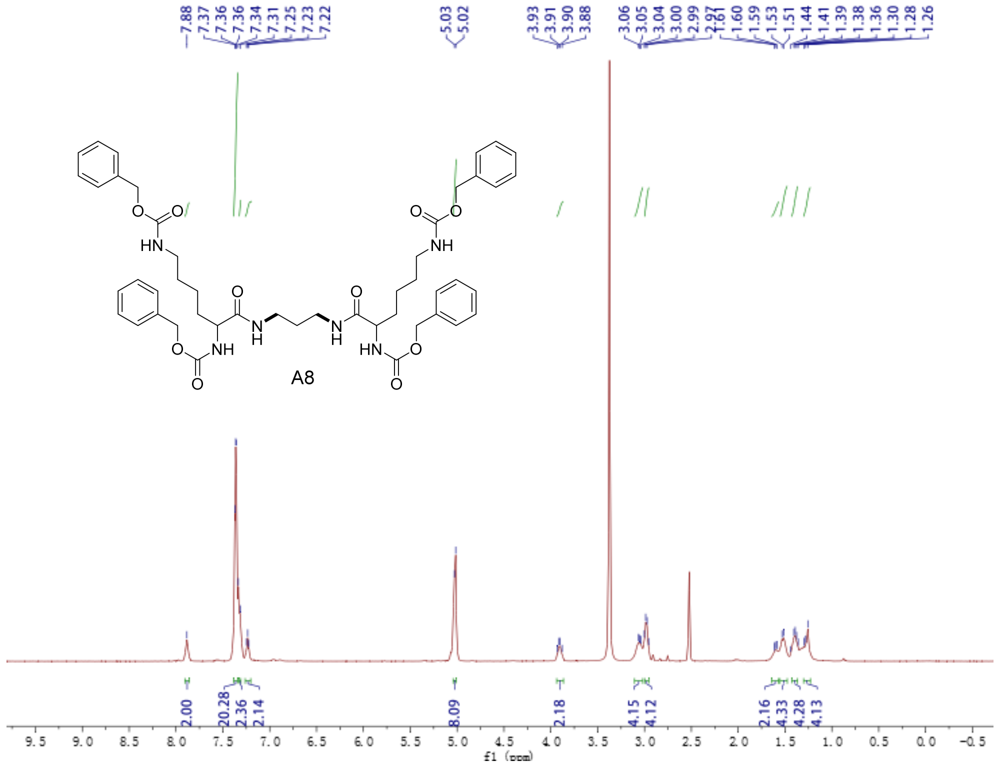

Figure S35. ^{1}H NMR spectrum (400 MHz, DMSO-d_6, 298K) of **A8**.

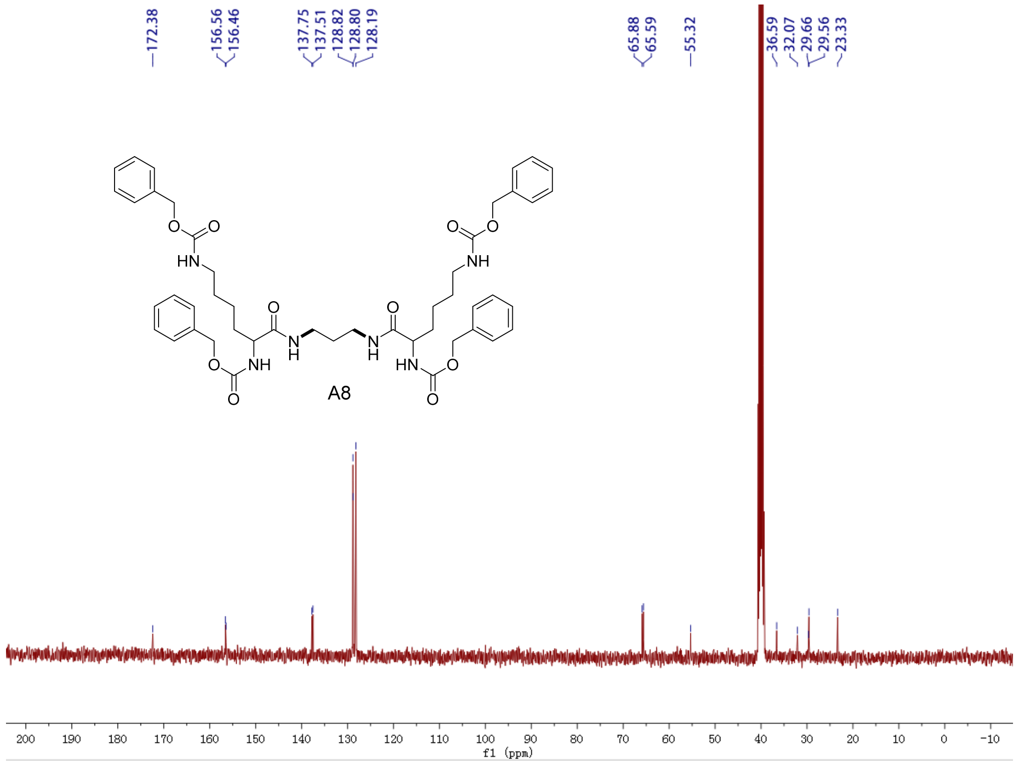

Figure S36. ^{13}C NMR spectrum (101 MHz, DMSO-d_6, 298K) of **A8**.

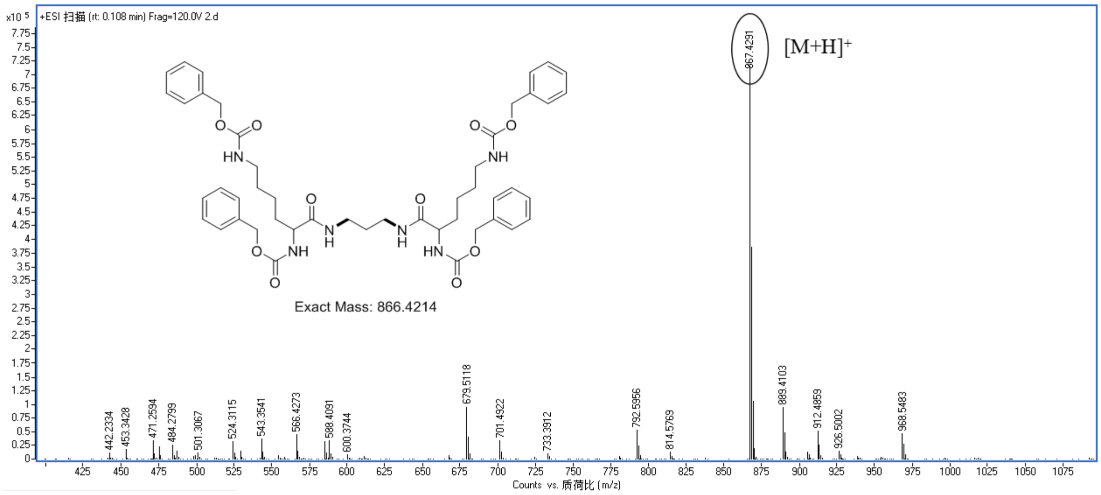

Figure S37. Mass spectrum of **A8**.

References

[1] KesavaRaju, C.S.; Pramanik, B.; Kar, T.; Rao, P.V.C.; Choudary, N.V.; Ravishankar, R. Low molecular weight gels: potential in remediation of crude oil spillage and recovery. *RSC Adv.* **2016**, 6 (58), 53415-53420.

[2] Vibhute, A.M.; Muvvala, V.; Sureshan, K.M. A sugar-based gelator for marine oil-spill recovery. *Angew. Chem. Int. Ed.* **2016**, 128 (27), 7913-7916.

[3] Prathap, A.; Sureshan, K.M. Organogelator-cellulose composite for practical and eco-friendly marine oil-spill recovery. Angew. Chem. Int. Ed. **2017**, 56 (32), 9405-9409.

[4] Ren, C.; Shen, J.; Chen, F.; Zeng, H. Rapid room-temperature gelation of crude oils by a wetted powder gelator. *Angew. Chem. Int. Ed.* **2017**, 56 (14), 3847-3851.

[5] Zhuan, C.; Li, Y.; Yuan, X.; Zhao, J.; Hou, X. A sorbitol-based phase-selective organogelator for crude oil spills treatment. *J. Appl. Polym. Sci.* **2018**, 136 (6), 47052.

[6] Majumder, L.; Chatterjee, M.; Bera, K.; Maiti, N.C.; Banerji, B. Solvent-assisted tyrosine-based dipeptide forms low-molecular weight gel: preparation and its potential use in dye removal and oil spillage separation from water. *ACS Omega* **2019**, 4 (11), 14411-14419.

[7] Sun, H.-L.; Chuai, J.; Wei, H.; Zhang, X.; Yu, H. Multi-functional organic gelator derived from phenyllactic acid for phenol removal and oil recovery. *J. Hazard. Mater.* **2019**, 366, 46-53.

[8] Pathak, N.P.; Yadav, S. A gelator-starch blend for dry powder based instant solidification of crude oil at room temperature. *Chem. Commun.* **2020**, 56 (20), 2999-3002.

[9] Damavandi, F.; Soares, J.B.P. Facile and efficient phase-selective powder polymer organogelator for oil spill remediation. *Langmuir* **2022**, 38 (41), 12666-12673.

[10] Yang, D.; Xia, S.; Bao, M.; Chen, X.; Kang, H.; Zhao, H.; Li, Y. A new class of amide-based organogels: from oil spill recovery to self-assembly structure analysis. *J. Mater. Chem. A* **2023**, 11 (12), 6181-6190.

[11] Abdellatif, M.M.; Sabry, M.N.; Ibrahim, S.; Bassem, S.M.; Khalil, W.K.B.; Abdel Gawad, F.Kh. Evaluation of mitigation role of L-phenylalanine-based low-molecular-weight

gelator against oil pollution-induced Nile tilapia toxicity. *Gels* **2023**, 9 (11), 848.

[12] Zhang, X.; Ma, K.; Yu, Z.; Zhou, J.; Zhang, C.; Dai, R. Reusable solid-form phase-selective organogelators for rapid and efficient remediation of crude oil spill. *Langmuir* **2024**, 40 (4), 2091-2101.

[13] Wang, L.; Zeng, Y.; Yin, R.; Zheng, Y.; Li, Z.; Zhou, L.; Yan, G.; Li, Z.; Chen, J.; Lai, J. Phase-selective organogelators with pH-triggered recovery for efficient oil spill treatment. *Langmuir* **2025**, 41 (21), 13482-13492.

[14] Googol, F.; Rahmati, A. Synthesis of dicholesteryl organogelator as a green sorbent nanomaterial for oil spill remediation. *Sci. Rep.* 2024, 14 (1), 21111.

[15] Zhang, W.; Du, Y.; Gao, Z.; Yu, F.; He, Y.P. Recyclable multifunctional PSOGs for rapid removal of wastewater pollutants (Oily and Dye). *Adv. Mater. Interfaces* **2025**, 12 (3), 2400525.

[16] Zainab; Zhang, L.J.; Zhang, C.C.; Yu, H. Reusable solid-form benzimidazole-based supramolecular organogelators for high efficiency oil spill recovery. *J. Mol. Struct.* **2025**, 1331, 141639.

[17] Pegu, A.O.; Moral, R.; Das, G. PSOG for selective thixotropic/thermo-reversible gelation in oil: Oil spill recovery and fuel-adulteration detection. *Chem. Eng. J.* **2026**, 539, 169128.
